# Progranulin from different gliocytes in the nucleus accumbens exerts distinct roles in FTD- and neuroinflammation-induced depression-like behaviors

**DOI:** 10.1186/s12974-022-02684-8

**Published:** 2022-12-29

**Authors:** Jing Wang, Simin Lai, Ting Zhou, Zhihao Xia, Weina Li, Wenqi Sha, Jingjie Liu, Yanjiong Chen

**Affiliations:** 1 Department of Immunology and Pathogenic Biology, College of Basic Medicine, Xi’an Jiaotong University Health Science Center, Xi’an, 710061 People’s Republic of China; 2 Department of Laboratory Medicine, The Second Affiliated Hospital, Xi’an Jiaotong University Health Science Center, Xi’an, People’s Republic of China; 3 Department of Neurology, The Second Affiliated Hospital, Xi’an Jiaotong University Health Science Center, Xi’an, People’s Republic of China

**Keywords:** Progranulin, NAc neuroinflammation, Neuroplasticity, p38 and NF-κB pathways, FTD-like behaviors, Depressive-like behaviors

## Abstract

**Background:**

Neuroinflammation in the nucleus accumbens (NAc) is well known to influence the progression of depression. However, the molecular mechanisms triggering NAc neuroinflammation in depression have not been fully elucidated. Progranulin (PGRN) is a multifunctional growth factor that is linked to the innate immune response and inflammation, and PGRN plays a key role in neurodegenerative diseases such as frontotemporal dementia (FTD). Here, the purpose of this study was to validate whether PGRN was involved in the NAc neuroinflammation-promoted depressive-like phenotype.

**Methods:**

A NAc neuroinflammation-relevant depression-like model was established using wild-type (WT) and PGRN-knockout (KO) mice after NAc injection with lipopolysaccharide (LPS), and various behavioral tests related to cognition, social recognition, depression and anxiety were performed with WT and PGRNKO mice with or without NAc immune challenge. RT‒PCR, ELISA, western blotting and immunofluorescence staining were used to determine the expression and function of PGRN in the neuroinflammatory reaction in the NAc after LPS challenge. The morphology of neurons in the NAc from WT and PGRNKO mice under conditions of NAc neuroinflammation was analyzed using Golgi–Cox staining, followed by Sholl analyses. The potential signaling pathways involved in NAc neuroinflammation in PGRNKO mice were investigated by western blotting.

**Results:**

Under normal conditions, PGRN deficiency induced FTD-like behaviors in mice and astrocyte activation in the NAc, promoted the release of the inflammatory cytokines interleukin (IL)-6 and IL-10 and increased dendritic complexity and synaptic protein BDNF levels in the NAc. However, NAc neuroinflammation enhanced PGRN expression, which was located in astrocytes and microglia within the NAc, and PGRN deficiency in mice alleviated NAc neuroinflammation-elicited depression-like behaviors, seemingly inhibiting astrocyte- and microglia-related inflammatory reactions and neuroplasticity complexity in the NAc via the p38 and nuclear factor of kappa (NF-κB) signaling pathways present in the NAc after neuroinflammation.

**Conclusions:**

Our results suggest that PGRN exerts distinct function on different behaviors, showing protective roles in the FTD-like behavior and detrimental effects on the neuroinflammation-related depression-like behavior, resulting from mediating astrocyte and microglial functions from the NAc in different status.

**Supplementary Information:**

The online version contains supplementary material available at 10.1186/s12974-022-02684-8.

## Introduction

Major depression is a debilitating psychiatric disorder [1], the incidence of which has increased during the COVID-19 pandemic [2], yet the underlying neurobiological mechanisms remain unknown. The nucleus accumbens (NAc) has been proposed to be a critical mediator of depression symptomatology, including reduced motivation and anhedonia [3, 4]. Studies in depressed patients and rodents after social defeat stress exposure found reduced NAc activation in response to reward-associated stimuli, along with evidence of lower volume in the NAc [5–7]. Deep brain stimulation of the NAc in treatment-refractory patients with depression can ameliorate anhedonia and exert antidepressant effects [8, 9]. In addition, medium spiny neurons (MSNs), the principal GABAergic cells in the NAc, have more stubby spine structures with smaller postsynaptic densities and an increase in the frequency of miniature excitatory postsynaptic currents (mEPSCs) in mice after social defeat [10]. Further studies indicated that the frequency of excitatory inputs is decreased in D1 (dopamine D1 receptor)-MSNs and increased in D2 (dopamine D2 receptor)-MSNs in mice displaying depression-like behaviors following chronic social defeat stress [11]. Importantly, the use of viral tracing approaches and chemogenetic or optogenetic tools demonstrated that NAc integration of different afferent excitatory and inhibitory inputs triggers emotional behaviors through mesolimbic and cortico-limbic circuits [4]. This local and global NAc function mediates depression-related outcomes, making it an attractive target for therapeutics.

Converging clinical and animal evidence implicates NAc inflammation in the pathogenesis of depression. In one clinical connectivity analysis, individuals who experienced the greatest deterioration in total mood after peripheral inflammation showed a highly significant reduction in the functional relationship between the subgenual anterior cingulate cortex and activity within the NAc, and this finding supports that of an animal study showing that immune stimulation-induced depression behavior in mice was accompanied by reduced neural activity in the NAc [12]. These results indicate that the mood-dependent modulation of NAc activity might underlie a potential mechanism for inflammation-induced modulation of hedonic tone. Moreover, several preclinical studies have further revealed that microglial activation and the resulting inflammation observed in the NAc are involved in the depressive-like behaviors induced by animal models of unpredictable chronic mild stress, high-fat/high-sucrose diets and systemic lipopolysaccharide (LPS) injection [13–15]. It is worth noting that the findings of our recent study echoed these data, demonstrating that NAc inflammation induced by direct bilateral NAc injection with LPS triggered depression-like behaviors in mice [16]. Taken together, our studies and those of others highlight a significant role for NAc inflammation in depressive-like disorders. However, how NAc neuroinflammation is produced and thereby influences depression is still not entirely understood.

Progranulin (PGRN) is a secretory glycoprotein consisting of 593 amino acid residues and is cleaved by serine proteases to granulin (GRN) [17, 18]. As a multifunctional growth factor, PGRN has been proven to exert a protective role in inflammation-related diseases, including rheumatoid arthritis [19, 20], inflammatory bowel disease [21], psoriasis [22] and asthma [23]. In the modulation of inflammation, PGRN mainly regulates TNF/TNFR-mediated proinflammatory signaling pathways by regulating extracellular regulated protein kinase 1 and 2 (ERK1/2), nuclear factor of kappa (NF-κB) and the expression of some chemokines, such as CXCL9 and CXCL10 [19, 24, 25]. Furthermore, PGRN maintains Treg function by affecting the Janus kinase/signal transducer and activator of transcription (JAK/STAT) pathway to reduce the cell response of T helper 1 (Th1) and Th17 cells and cytokine secretion [26] and induce IL-10 generation through the forkhead box protein O4/signal transducer and activator of transcription 3 (Foxo4/STAT3) signaling pathway [27]. Conversely, PGRN has detrimental effects when it is cleaved to yield discrete fragments, named GRN. For example, GRN assists in recruiting CpG oligonucleotides in macrophages by binding to Toll-like receptor 9 to exacerbate systemic lupus erythematosus severity [28, 29], and GRN stimulates secretion of proinflammatory interleukin (IL)-8 by epithelial cells in multiple sclerosis [30, 31]. In general, PGRN functions as a double-edged sword by playing proinflammatory and anti-inflammatory roles in immune-mediated conditions, and the roles played may rely on the pathology and physiology of the diseases.

With regard to the central nervous system, it is well established that PGRN is expressed in neurons and microglia [32] and plays important roles in lysosomal function, neuronal survival and function and neuroinflammation and as a therapeutic target in various neurological disorders including traumatic brain injury, Alzheimer’s disease, Parkinson’s disease and Huntington’s disease [33–35]. Frontotemporal dementia (FTD), as an early-onset neurodegenerative disease, is the second most common form of dementia and manifests clinically with progressive behavioral and personality disturbances including disinhibition, perseveration and emotional blunting, evolving into generalized cognitive impairment and eventual death, and mutations in PGRN has been proven to be a main cause during this process [36, 37]. Notably, FTD patients with a *GRN* variant and PGRN-deficient mice have been reported to display depression-like behaviors [38, 39], indicating that PGRN may be involved in the development of depression. A better understanding of PGRN activities and mechanism of action will lead to new insights into brain function in depression and may facilitate the development of new therapies for depressive disorders.

In this context, we hypothesized that PGRN regulates neuroinflammation occurring in the NAc and influences the NAc inflammation-induced depression-like phenotype. To test this hypothesis, using LPS injection into a bilateral NAc-induced inflammation mouse model, we examined the possible involvement of PGRN in NAc neuroinflammatory reactions and thereby depressive-like behaviors. Furthermore, we focused on the possible mechanism of PGRN in the neuroinflammatory response occurring in the NAc.

## Materials and methods

### Animals

Male wild-type (WT) mice aged 7 weeks were purchased from Vital River Laboratory Animal Technology Co., Ltd. (Beijing, China). PGRN-knockout (PGRNKO) mice on a C57BL/6 background were obtained from Professor Cao (Chongqing Medical University), and the generation and genotyping of PGRNKO mice was based on the Cao laboratory's protocol for all experiments. All mice were housed individually in a cage and given free access to standard food and water in a temperature-controlled room. The room conditions were set at a temperature 23 ± 2 °C with 50 ± 10% humidity and a standard 12-h light–dark cycle (lights on 7:00 am–7:00 pm). All animals were allowed to acclimate to the facility for one week prior to the start of any experiments. Mice were randomly allocated to the different experimental groups, and experiments and data acquisition were performed in a blinded and unbiased fashion. All experimental procedures were performed in compliance with the National Institutes of Health guidelines for the use of experimental animals and were approved by the Animal Ethics Committee of Xi’an Jiaotong University.

### Stereotaxic surgery

Mice were anaesthetized with isoflurane (4.0% for induction and 1.0% thereafter for maintenance) and their heads were fixed in a stereotaxic frame (RWD Life Science Co., Shenzhen, China). The skull surface was exposed and all skull measurements were made relative to bregma. Drugs injection into the NAc was performed using a microinjection needle with a 10-μl microsyringe (Hamilton, USA) to deliver at a rate of 0.5 µl/min using an automated injection pump (KDS Legato™ 130, USA). After the infusion was completed, the microsyringe was left in place for 10 min to allow for diffusion of drugs. The stereotaxic coordinates for NAc injection were anterior posterior (AP) 1.50 mm, medial lateral (ML) 1.00 mm and dorsal ventral (DV) 4.50 mm relative to bregma. WT or KO mice were bilaterally injected into the NAc with 0.5 µl saline or LPS (100 ng). To further implicate the role of PGRN in LPS-induced mice, 0.5 µl of PGRN (500 ng) was bilaterally injected into the NAc 30 min prior to saline or LPS administration in PGRNKO mice. The dosage of LPS (100 ng) were selected based on preliminary experiments to achieve a balance between the enhancement of drug activities and minimal potential side effects. The dose of PGRN (500 ng) was selected based on previous studies showing the beneficial effects of these doses in animal models of memory impairment and anxiety, traumatic brain injury and acute kidney injury [40–42].

### Immunofluorescence staining

Mice were anesthetized with sodium pentobarbital solution (100 mg/kg) and killed by cervical dislocation at 24 h after injection. The NAc was rapidly collected and stored at − 80 °C after being embedded in OCT. The brain tissue was sectioned coronally into 10-µm-thick sections using an ultramicrotome. The sections were blocked for 45 min with 10% rabbit serum or 10% donkey serum solution. Subsequently, brain tissue slices were incubated overnight with a mixture of primary antibodies specific for NeuN (1:400; Abcam, USA), GFAP (1:300; Cell Signaling, USA) or Iba1 (1:200; Servicebio, China) and PGRN (1:50; R&D Systems, USA) at 4 °C. The slices were then washed and incubated with FITC-labeled IgG (1:500; Bioss; green fluorescence) and Cy3-labeled IgG (1:500; Bioss; red fluorescence) for 1 h at room temperature. Finally, after 10 min of incubation with DAPI, the stained sections were examined under a fluorescence microscope (Carl Zeiss, Axio Scope A1, Germany). To explore the correlation between NeuN-FITC, GFAP-FITC or Iba1-FITC and PGRN-Cy3, the number of immunoreactive cells in a 10× microscopic field was observed.

### Behavioral experiments

All tests were carried out during the light phase (between 9:00 am and 4:00 pm), and the mice were allowed to acclimatize to the behavioral test room for at least 1 h. Eight or twelve mice per group were used in the behavioral experiments. All behavioral experiments were performed by observers who were blinded to the experimental groups.

#### Novel object recognition

Novel object recognition tests were performed according to a previous report [43]. The mice were individually habituated to an empty box (30 × 45 × 16 cm high) for 10 min on Day 1 and Day 2. Mice were trained in 5-min trials, and this trial was repeated three times at 15-min intervals on Day 3. During the training phase, two identical objects (A and B) were placed in the middle area of the chamber. Each animal was then placed in the box and allowed to freely explore the area for 5 min. Three hours after the training phase, one object (A or B randomly) was replaced by a novel object (C), and exploratory behavior was again evaluated for a total of 5 min in test 1. In test 2, performed 24 h after test 1, object C was replaced by another novel object (D). The relatively immovable objects made of wood and varied in shape and texture were children's toys (Lego pieces), which were not larger than the mice. During the trials, the open field box and all objects were thoroughly cleansed between sessions using 75% ethanol to prevent odor recognition. Exploration was defined as sniffing or touching the object with the nose and/or forepaws. The discrimination of visual novelty was assessed by a recognition index, defined as follows: time of novel object exploration/(time of novel object exploration + old object exploration time) × 100%. Smart 3.0 software was used to record the exploration time. Animals displaying a dramatic bias for the objects during training (> 65% investigation of one object) were excluded from the data pool.

#### Y maze

The Y maze was a Y-shaped apparatus with three arms (30 cm long, 6 cm wide, and 15 cm high). The arms were at a 120° angle from each other. The test was performed in 2 phases: phase 1, with one of the lateral arms closed, and phase 2, with all three arms open. In phase 1, mice were randomly placed at the end of one of the 2 arms and allowed to explore for 3 min. Mice were again randomly placed at the end of one of the 3 arms of the maze 2 h following phase 1. In phase 2, the mazes’ three arms were open for exploration for 3 min. The indices were defined as follows: entries of novel arm exploration/(entries of novel arm exploration + entries of old arms exploration) × 100%; time of novel arm exploration/(time of novel arm exploration + time of old arms exploration) × 100%.

#### Morris water maze

The Morris water maze protocol was based on a previous study [44]. The water surface was divided into four quadrants, each with an entry point. Signs of the same size and color, but in different shapes, were pasted in the middle of the water tank wall in each quadrant. The experiment was divided into a spatial learning phase (four trials a day for 4 or 5 days) and a space exploration phase (24 h after the last trial). During the spatial learning phase, a plexiglass platform with a diameter of 10 cm was hidden 1–1.5 cm below the water surface in the second quadrant. All mice were permitted to acclimatize to the environment of the room for 1 day before the learning phase. At the beginning of each experiment, mice were placed randomly in the water facing the tank wall at one of four fixed entry points. The time limit for each mouse to find the platform was 60 s. If the mice failed to find the platform within 60 s, the mice were manually placed on the platform for 15 s. If the mice climbed onto the platform within 60 s and stayed on there for 10 s, it was considered that the mouse had learned to find the platform. After 10 s, the mice were returned to their home cages. A camera was placed above the tank and was used to automatically track each animal’s performance with Smart 3.0 software. After each trial, the mice were wiped with a towel and placed in their home cage for at least 10–15 min until the next training session. The time from when the mice were put into the pool to the time that the mice successfully found the platform was recorded as the latency period of a single training platform climbing. The average value of the platform-climbing latency in four trials per day was set as the key index for the learning ability of the mice. The platform was removed during the space exploration phase. Mice were placed in water from the entry point farthest from the platform and recorded for 60 s. During this phase, the spatial memory retention ability was represented by the number of platform site crossings, the time in the target quadrant, and the time in the target.

#### Social interaction test

The social interaction test was performed using the method previously described by Ni et al. [43]. The test apparatus consisted of a 43 × 43 cm open field with 2 plastic containers (A and B, 8-cm-diameter plastic cylinders with holes for odor interaction) in 2 opposite corners. Chamber A contained a probe mouse (8-week-old adult C57BL/6J), while chamber B remained empty. The social zone was defined as the 8-cm area around chamber A, and the nonsocial zone was defined as the 8-cm area around chamber B. The experimental mice were placed in the center of the open field for 5 min of exploration. Experimental mice in different regions were recorded and analyzed using Smart 3.0 software. The social interaction index was defined as follows: time spent in social zone/ total time in both zones. After each test, the observation chamber was cleaned with 75% ethanol to remove residual odor.

#### Elevated plus maze

The elevated plus maze was made of wood, painted black, and had two open arms (25 × 5 cm) and two enclosed arms of the same size with 15-cm high walls. The apparatus was elevated 55 cm above the floor. Each mouse was placed individually in the central square of the EPM apparatus and allowed to explore freely for 5 min. Each mouse was recorded and analyzed using the same system as described above. The parameters recorded were the number of entries into the open and closed arms and the time spent exploring the open and closed arms. The index was defined as follows: entry into open arms (%) = the number of entries into open arms/(the number of entries into open arms + the number of entries into closed arms); time in open arms (%) = time in open arms/(time in open arms + time in closed arms).

#### Sucrose preference test

To quantify inflammation-induced depression-like behavior, we subjected mice to the two-bottle sucrose preference test (SPT). Approximately 2 weeks prior to treatment, mice were trained by simultaneous presentation with a bottle of water and a bottle of 1% (wt/vol) sucrose solution. The bottle was weighed before the lid was placed on each mouse's home cage and was reweighed to determine the amount of sucrose solution and water consumed after 24 h. The positions of the bottles were changed every 12 h to ensure that the mice did not develop a preference for one side. Mice were trained until a stable baseline preference was established, and then treatments were administered. After saline or LPS injection, water and sucrose consumption was measured in WT and KO mice for 24 h. Sucrose preference was calculated as the percentage of sucrose solution consumed relative to the total fluid intake: sucrose intake/(sucrose intake + water intake) × 100%.

#### Tail suspension test

The tail suspension test (TST) was performed on WT and PGRNKO mice 24 h after injection and was conducted according to our previous study [15]. A mouse was suspended 30 cm above the floor by hanging the mouse on the anchor hook for 10 min using a small piece of tape placed approximately 2 cm from the tip of the tail. The duration of immobility over the 10 min was recorded and calculated.

#### Forced swimming test

The forced swimming test (FST) was performed 2 h after the TST. The mice were placed in clear plastic cylinders (30 cm high × 20 cm diameter), which were partially filled to 15 cm with 24 ± 1 °C water, for 6 min. The water in the cylinder was changed between each mouse during the test sessions. The test duration was video recorded for 6 min, and the immobility time of the mice was calculated for the final 4 min.

### Quantitative real-time PCR

The NAc was dissected bilaterally on ice and immediately frozen in sample tubes placed in liquid nitrogen. The tissue was stored frozen at − 80 °C until processing. Next, total RNA was isolated using the TRIzol^®^ RNA extraction kit (Invitrogen). Reverse transcription was performed using 1 µg of total RNA for each sample by using the PrimeScript^TM^ RT reagent kit (Takara Bio Inc.) according to the manufacturer’s instructions. Real-time PCR amplification was performed using the Stratagene Mx3005p Real-Time PCR Detection System (Agilent Technologies, Santa Clara, CA, USA) with SYBR Green master mix (Takara Bio Inc.) in a final volume of 20 µL that contained 1 µl of cDNA template from each sample. The sequences of the forward (F) and reverse (R) primers are shown in Table 1. The PCR protocol was as follows: an initial denaturation at 95 °C for 30 s, 40 cycles at 95 °C for 5 s and 60 °C for 30 s, and one cycle at 95 °C for 1 min, 55 °C for 30 s, and 95 °C for 30 s. After the completion of the reaction, specificity was verified by melting curve analysis. The relative mRNA values were normalized to the control values of the *GAPDH* gene and calculated using the comparative cycle threshold (△△Ct) method [45].

**Table 1:** The sequence of primer pairs for PCR analysis

Mice genes	Primer sequence (5′ → 3′)	Length (bp)
PGRN	F: GGTTGATGGTTCGTGGGGATGTTG	185
	R: AAGGCAAAGACACTGCCCTGTTGG	
Iba1	F: CGATATTATGTCCTTGAAGCGAAT	140
	R: CCAGCATCATTCTGAGAAAGTC	
GFAP	F: ATCTATGAGGAGGAAGTTC	119
	R: TATTGAGTGCGAATCTCT	
NLRP3	F: AGAAGAGTGGATGGGTTTGCT	126
	R: GCGTTCCTGTCCTTGATAGAG	
ASC	F: GTCACAGAAGTGGACGGAGTG	104
	R: CTCATCTTGTCTTGGCTGGTG	
Caspase-1	F: CGTGGAGAGAAACAAGGAGTG	192
	R: AATGAAAAGTGAGCCCCTGAC	
PSD-95	F: ATCCTGTCGGTCAATGGTGTT	259
	R: AGTCCTTGGTCTTGTCGTAGTC	
SYP	F: TGCCAACAAGACGGAGAGT	142
	R: CGAGGAGGAGTAGTCACCAA	
BDNF	F: TACCTGGATGCCGCAAACAT	185
	R: AGTTGGCCTTTGGATACCGG	
GAPDH	F: TGTGTCCGTCGTGGATCTGA	150
	R: TTGCTGTTGAAGTCGCAGGAG	

### Enzyme-linked immunosorbent assay (ELISA)

The supernatants obtained from NAc tissue were collected immediately. The concentrations of the cytokines related to inflammation, including IL-1β, TNF-α, IL-6 and IL-10, were detected by ELISA kits according to the manufacturer’s instructions (Invitrogen, Thermo Fisher). Sample values were read from the standard curve. Each sample was assayed in duplicate.

### Western blot analysis

Western blotting was performed as we described previously [46]. Briefly, NAc tissue was immediately collected after the behavioral tests and lysed in RIPA lysis buffer (1× phosphate-buffered saline, 1% Nonidet P-40, 0.5% sodium deoxycholate, and 1% sodium dodecyl sulfate; Beyotime, Shanghai, China) containing a 1:50 ratio of protease and phosphatase inhibitor cocktail (Roche, Basel, Switzerland). The homogenates were incubated on ice for 20 min and then centrifuged at 12,000×*g* for 15 min at 4 °C. Protein samples were assayed using a BCA kit (Solarbio Science & Technology Co., Ltd., Beijing, China), separated by sodium dodecyl sulfate‒polyacrylamide gel electrophoresis (SDS‒PAGE), and transferred to a polydimer vinyl fluoride (Millipore, USA) membrane. After incubation in a blocking solution of 5% skim milk in Tris-buffered saline containing 0.1% Tween 20 for 2 h at room temperature, the membrane was probed overnight at 4 °C using the primary antibodies. The following primary antibodies were used: anti-GAPDH (1:2000; Proteintech, USA), anti-phosphorylated ERK1/2 (p-ERK1/2, 1:1000; CST, USA), anti-ERK1/2 (ERK1/2, 1:1000; CST, USA), anti-phosphorylated JNK (p-JNK, 1:1000; CST, USA), anti-JNK (1:1000; CST, USA), anti-phosphorylated P38 (p-P38, 1:1000; CST, USA), anti-P38 (1:1000; CST, USA), anti-phosphorylated P65 (p-P65, 1:1000; CST, USA), and anti-P65 (1:1000; CST, USA). This incubation step was followed by incubation with horseradish peroxidase (HRP)-coupled anti-rabbit IgG or HRP-coupled anti-mouse IgG for 1 h at room temperature. Proteins were visualized by incubation in ECL solution (Millipore, USA), and images were captured using a Fusion FX5 camera system. The scanned images were measured using ImageJ software (National Institute of Health, Bethesda, MA, USA). Specific bands were then quantified and normalized to the GAPDH loading control for each lane and each blot.

### Golgi–Cox staining

The brain was carefully extracted and fixed in paraformaldehyde fixative for more than 48 h. Then, the brain was cut into 2–3 mm thick tissue blocks according to the location of the NAc and washed with saline. Then, the samples were immersed in 20 ml Golgi–Cox solution and stored at room temperature for 14 days in the dark (the solution was changed every three days). After 3 immersions in distilled water, 80% glacial acetic acid was poured onto the tissue to immerse the tissue overnight. The tissue was washed with distilled water and placed in 30% sucrose after the tissue became soft. Tissues were cut into 100-μm sections with a vibrating microtome, mounted on gelatin slides and air-dried overnight in the dark. The air-dried tissue slides were treated with concentrated ammonia water for 15 min, washed with distilled water for 1 min, treated with acid fixative solution for 15 min, washed with distilled water for 3 min, air-dried, and sealed with glycerin gelatin. Finally, microscopic examination and image acquisition analysis were performed. Sholl analysis as well as determinations of the length of dendritic spines, the number of dendritic spines, and dendritic spine density was performed with ImageJ. Dendritic spine density was quantified as the number of protrusions on dendritic branches per 10 μm dendritic length.

### Statistical analysis

All data are presented as the mean ± SEM. Data were analyzed using IBM SPSS Statistics 20.0. Student’s *t* tests, one-way and two-way analysis of variance (ANOVA) were performed as appropriate. After ANOVA was performed, the least significant difference (LSD) post hoc test was used for multiple comparisons. *p* values less than 0.05 were considered statistically significant.

## Results

### Expression and distribution of PGRN in the NAc following NAc neuroinflammation

**Fig. 1:**
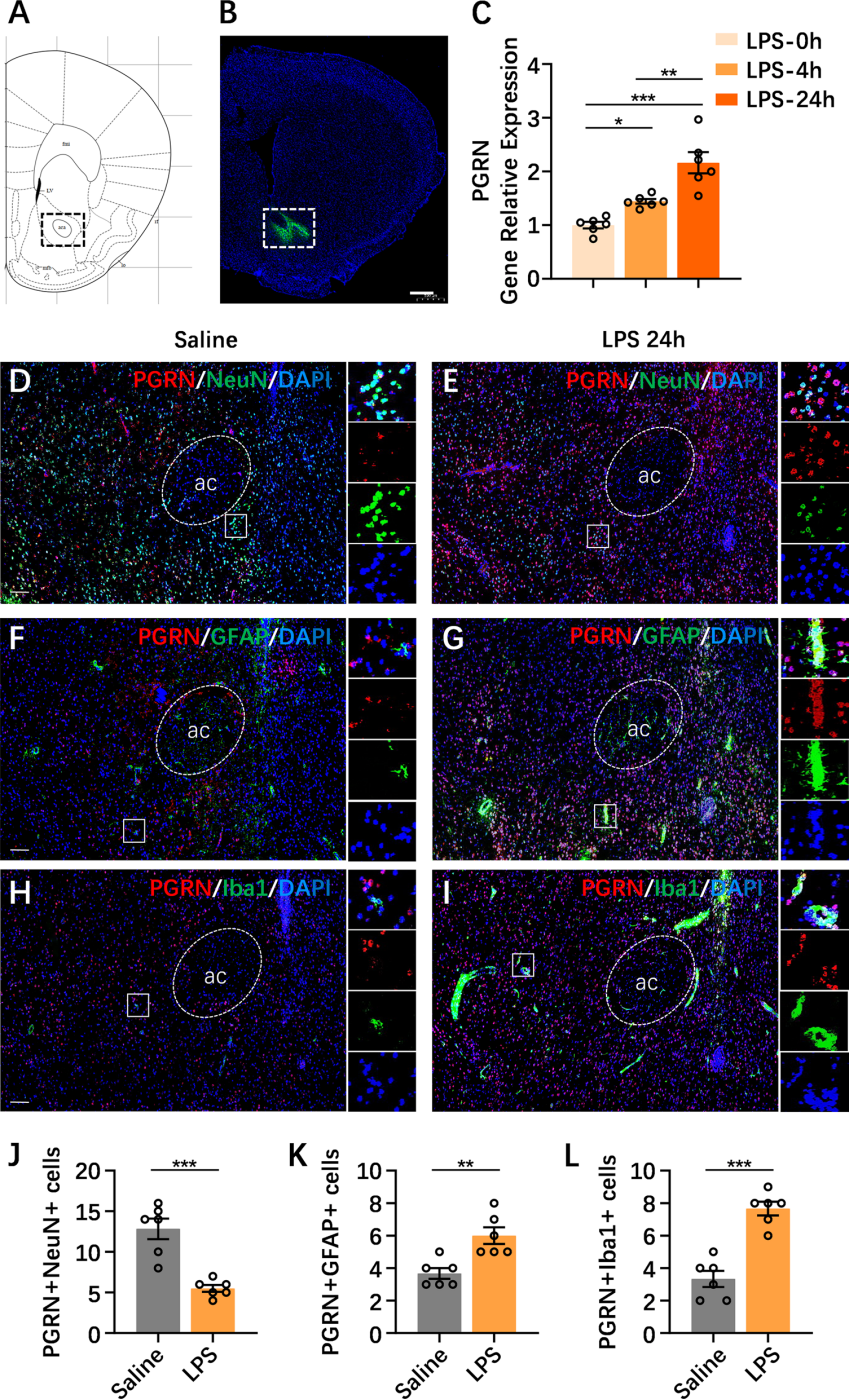
Expression and distribution of PGRN in the NAc following NAc neuroinflammation. **A**, **B** Representative immunofluorescence images showing the exact site and diffusion degree after NAc nuclei injection (scale bar 500 μm). **C** RT-PCR analysis showed PGRN mRNA was increased 4 h after NAc LPS injection, and its expression was continuously and drastically enhanced 24 h after NAc LPS administration compared to that after control treatment. **D**, **E** A large of PGRN was located at NeuN-labeled neurons in the NAc under normal circumstance, but decreased PGRN expression in the neurons from NAc was observed during NAc LPS challenge. **F**, **G** Compared to sporadic scattered of PGRN was expressed in the GFAP-labeled astrocytes in the NAc from NAc saline-treated mice, NAc LPS exposure obviously increased PGRN expression in astrocyte of NAc in mice. **H**, **I** Likewise, distribution of PGRN in the Iba1-labeled microglia from the NAc was significantly increased after NAc LPS exposure, when compared with the scarcely any PGRN expression in the microglia of NAc from NAc saline-treated mice. **J–L** The statistical numbers of co-localized between NeuN-labeled neurons, GFAP-labeled astrocyte or Ibai-labeled microglia and PGRN-positive cells in the NAc in NAc saline-treated and NAc LPS-treated mice. NeuN, marker of neurons; GFAP, marker of astrocytes; Iba1, marker of microglia; nuclei, stained with DAPI (blue); scale bar: 100 µm (**D**–**I**). *n* = 6 per group. Data were analyzed by Student’s *t*-test, and expressed as mean ± SEM. *p < 0.05, **p < 0.01, ***p < 0.001

To assess the potential role of PGRN in NAc neuroinflammation, we evaluated PGRN mRNA expression in the NAc in a mouse model of NAc inflammation triggered by direct LPS microinjection in the NAc as described previously [16] (the exact site and diffusion degree after NAc nucleus injection are presented in Fig. 1A and B). As shown in Fig. 1C, PGRN mRNA was increased 4 h (*p* = 0.021) after NAc LPS injection, and its expression was continuously and drastically enhanced 24 h after NAc LPS administration (*p* = 0.0001) compared to that after control treatment, indicating that NAc neuroinflammation led to PGRN mRNA in the NAc being upregulated in a time-dependent manner. Next, we identified the distribution pattern of PGRN protein in NAc neurons and gliocytes under both normal conditions and the condition of NAc neuroinflammation induced by 24 h after NAc injection with LPS. We observed that most PGRN was well colocalized with NeuN (12.83 ± 3.13), a marker of neurons (Fig. 1D, J), and sporadically scattered PGRN was expressed in GFAP-labeled astrocytes (3.67 ± 0.82; Fig. 1F, K) and Iba1-labeled microglia (3.33 ± 1.21; Fig. 1H, L) in the NAc in the saline-control treatment. Twenty-four hours after NAc injection with LPS, PGRN protein levels in the NAc were remarkably elevated compared to those in saline-control mice, similar to the PGRN mRNA alterations induced by NAc LPS injection; importantly, we observed that PGRN expression in the NeuN-labeled neurons (5.50 ± 1.05) of the NAc was obviously decreased (*p* = 0.0003; Fig. 1E, J), whereas PGRN was distributed in GFAP-labeled astrocytes (6.00 ± 1.26; *p* = 0.004; Fig. 1G, K), and Iba1-labeled microglia (7.67 ± 1.03) within the NAc (*p* = 0.0001; Fig. 1I, L) were moderately increased when compared with those of the control group. Thus, PGRN was mainly expressed in the neurons of the NAc under normal circumstances, while PGRN was colocalized with the neurons, astrocytes and microglia of the NAc after LPS injection, suggesting that PGRN expressed in the neurons, astrocytes and microglia of the NAc possibly has an impact on the NAc inflammation-induced depressive-like phenotype.

### PGRN deficiency in the NAc plays an antidepressant role in the depressive-like phenotype induced by NAc neuroinflammation

**Fig. 2:**
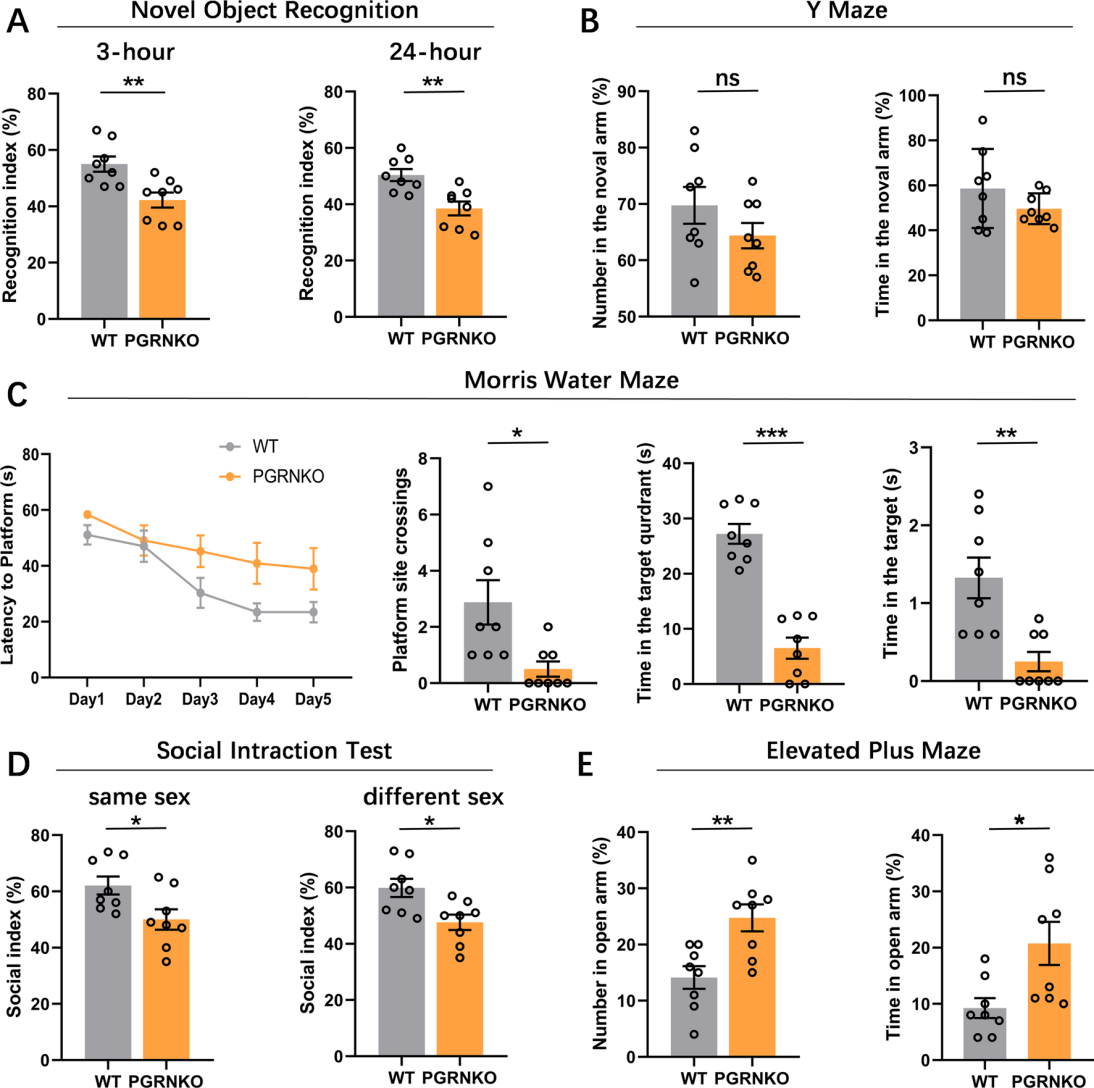
PGRN deficiency induces FTD-like behaviors. **A** PGRN deficiency decreased the recognition index relative to WT control group in novel object recognition test. **B** PGRN deficiency did not change the alteration triplet in the Y maze, compared to WT mice. **C** Compared with WT mice, PGRN deficiency in mice had no effects on the latency the platform, but its deficiency decreased the platform site crossings, time in the target quadrant and time in the target in morris water maze test. **D** PGRN deficiency decreased social interaction changes in both same sex and different sex, compared to WT mice. **E** PGRN deficiency in mice were disinhibited due to they spent more time and numbers in open arms than their WT littermates in the elevated plus maze test. *n* = 8 per group. Data were analyzed by Student's *t*-test, and expressed as mean ± SEM. *p < 0.05, **p < 0.01, ***p < 0.001, ns not significant

In our present study, we first examined a battery of FTD-like behaviors relevant to psychiatric symptoms in PGRNKO mice at 2 months, such as cognition, social interaction and disinhibition. Our results showed that PGRN deficiency induced short-term (*p* = 0.005) and long-term (*p* = 0.003) memory cognitive impairment in mice in the Novel object recognition test (Fig. 2A) but did not alter the recognition index in the Y maze test (*p* = 0.197, *p* = 0.199; Fig. 2B) compared to WT mice. Consistently, PGRN deficiency also impaired the spatial memory of mice in the Morris water maze test, as shown in Fig. 2C. The latency of mice climbing the platform showed no obvious changes when the two groups were compared during the training stage (*p* = 0.197). In the probe test, PGRNKO mice displayed fewer platform site crossings (*p* = 0.013) and spent less time in the target quadrant (*p* = 0.0001) and target (*p* = 0.002) than WT mice. In addition, PGRNKO mice showed decreased social interaction in both same-sex (*p* = 0.025) and different-sex (*p* = 0.012) social interaction tests (Fig. 2D), and PGRNKO mice were disinhibited because they spent more time in (*p* = 0.016) and had more entries into (*p* = 0.004) the open arms than their WT littermates in the elevated plus maze test (Fig. 2E). Together, these behavioral results demonstrate that PGRNKO mice at 2 months display FTD-like behaviors, showing increased disinhibition-like behaviors and deficits in social interaction and cognition.

**Fig. 3:**
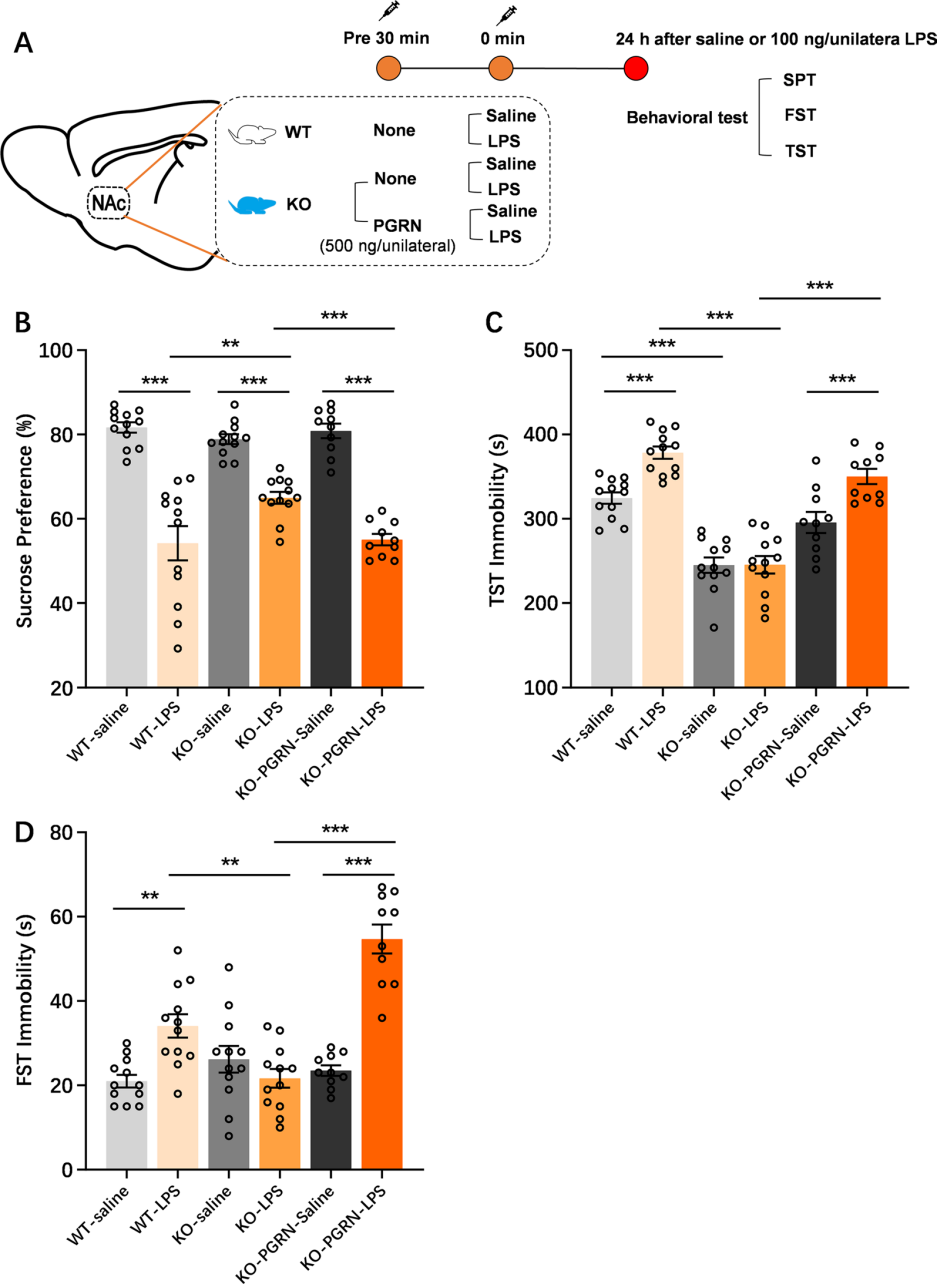
PGRN deficiency in the NAc plays an antidepressant role in the NAc LPS challenge-induced depressive-like phenotype. **A** Graphical depiction of the experimental timeline in depression-like relevant behavioral tests after NAc LPS challenge in the WT and PGRNKO mice. **B** Sucrose preference between WT saline-control mice and PGRNKO saline-control mice had no obvious changes. PGRN deficiency in mice attenuated the decreased of sucrose preference induced by NAc LPS exposure, compared to NAc LPS-treated WT mice. NAc Pretreatment with recombinant PGRN decreased the sucrose preference increased in the PGRNKO mice after NAc LPS injection, compared to control group. **C** Immobility time of TST in NAc saline-treated PGRNKO mice was marked less than that of NAc saline-treated WT mice. NAc LPS exposure-induced TST immobility increased was reversed in the PGRNKO mice, while recombinant PGRN pretreatment enhanced the decreased in TST immobility in the PGRNKO mice after NAc LPS injection. **D** There are no significant in FST immobility between WT saline-treated and PGRN saline-treated mice. But, PGRN deficiency in mice reversed the NAc LPS challenge-induced an increase of FST immobility relative to NAc LPS-treated WT mice. Recombinant PGRN injection into NAc significantly elevated the decreased in the FST immobility from PGRNKO mice during NAc LPS exposure, compared to control group. FST: forced swim test, TST: tail suspension test. *n* = 10–12 per group. Data were analyzed by one-way ANOVA followed by the LSD multiple comparisons test, and expressed as mean ± SEM. **p < 0.01, ***p < 0.001, ns not significant

Our published study proved that NAc inflammation induced by NAc injection with LPS is involved in depression development [16]. In the current study, we attempted to determine whether PGRN affected the NAc neuroinflammation-induced depressive-like phenotype by using global PGRNKO mice. The experimental procedure of the behavioral tests is depicted in Fig. 3A. In the present data (Fig. 3B–D), we did not observe any statistical significance in FST immobility (*p* = 0.196) or sucrose preference (*p* = 0.400) between NAc saline-treated WT and PGRNKO mice, but the immobility time in the TST in NAc saline-treated PGRNKO mice was markedly less than that in NAc saline-treated WT mice. These results indicate that PGRNKO mice did not exhibit altered depression-like behaviors at baseline. However, NAc neuroinflammation induced at 24 h following NAc LPS exposure triggered a series of depressive-like behaviors in WT mice, such as an obvious elevation in immobility time in the FST (*p* = 0.001) and TST (*p* = 0.0001) and a decline in preference for sucrose (*p* = 0001), which were abrogated in PGRNKO mice after NAc LPS injection (sucrose preference, *p* = 0.002; TST, *p* = 0.0001; FST, *p* = 0.003; Fig. 3B, C). It is likely that PGRN deficiency alleviates inflammatory-induced depressive-like behaviors in the NAc. To further clarify the essential role of PGRN expressed in the NAc in the NAc neuroinflammation-induced depression-like phenotype, we performed a rescue experiment on PGRNKO mice in which recombinant PGRN was injected into the NAc before LPS administration and then mice were subjected to various depressive-associated behavioral tests. The behavioral experiments showed that the injection of PGRN in the NAc reversed the antidepressant effects on NAc inflammation-induced depressive-like behaviors in PGRNKO mice, which displayed depressive-like symptoms (Fig. 3B–D), revealed by immobility latency increases in the FST (*p* = 0.0001) and TST (*p* = 0.0001) and a decline in sucrose preference (*p* = 0.0001). Moreover, PGRN pretreatment in the NAc showed no obvious changes in NAc saline-treated PGRNKO mice compared to control saline-treated WT mice. Collectively, these results demonstrate that PGRN deficiency in the NAc plays an antidepressant role in the development of the NAc inflammation-associated depressive-like phenotype.

### PGRN deficiency alleviates the NAc neuroinflammatory responses induced by NAc injection with LPS

**Fig. 4:**
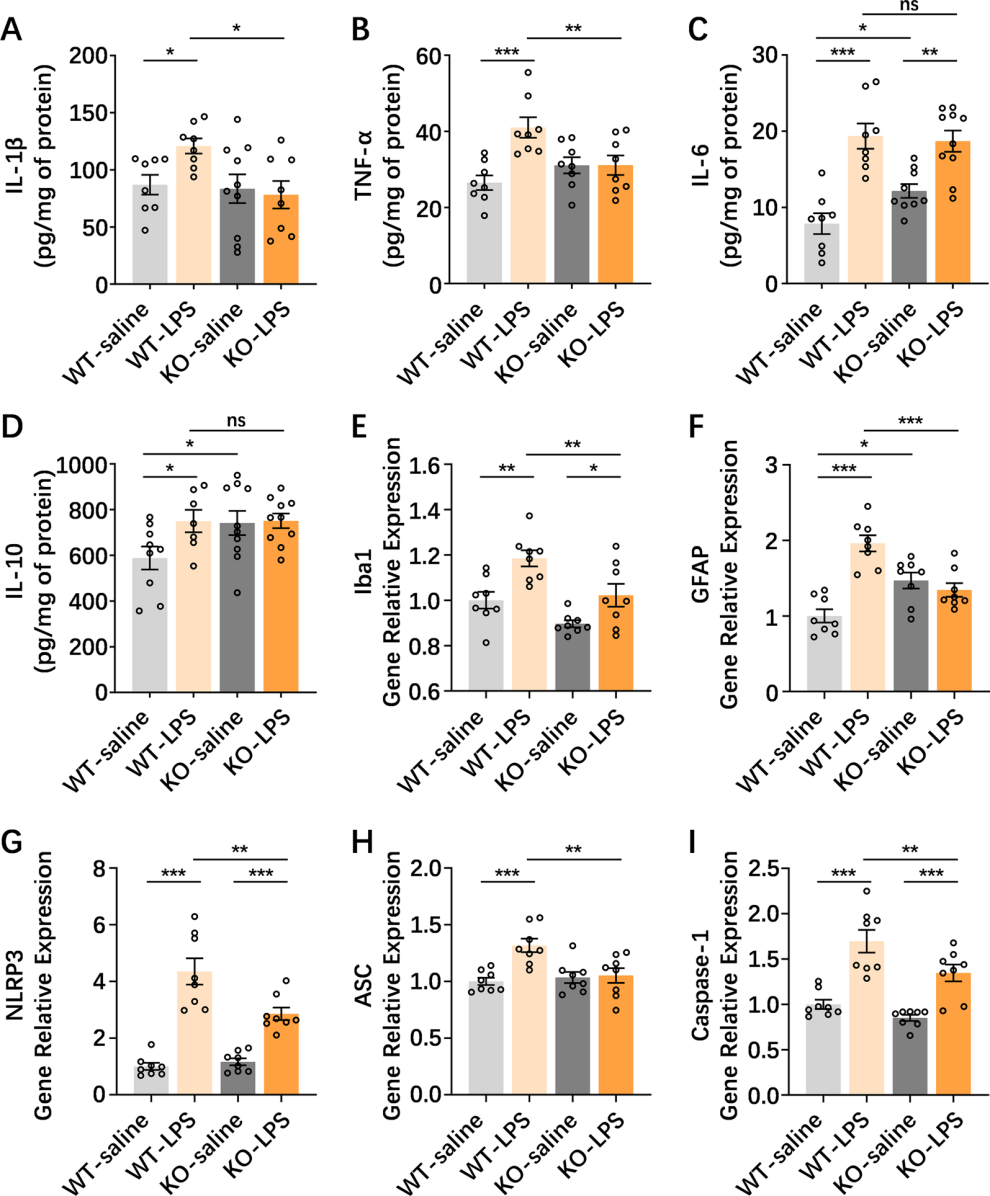
PGRN deficiency alleviates NAc neuroinflammatory responses induced by NAc LPS injection. **A**–**D** ELISA analysis demonstrated that NAc saline-treated PGRNKO mice exhibited slightly enhanced expression in IL-6 and IL-10 but no changes in IL-1β and TNF-α in the NAc, compared to NAc saline-treated WT mice. PGRN deficiency reduced an increase level of IL-1β and TNF-α in the NAc triggered by NAc injection of LPS, whereas did not alter elevated expression in the IL-6 and IL-10 after NAc LPS injection, compared to NAc LPS-treated WT mice. *n* = 8–10 per group. **E**, **F** RT-PCR analysis showed that GFAP-labeled astrocyte mRNA in the NAc from saline-treated PGRNKO mice was increased relative to the saline-treated WT mice. NAc LPS exposure promoted astrocyte and microglia mRNA expression elevation in WT mice, but these alterations was abolished in the NAc LPS-treated PGRNKO mice. *n* = 8 per group. **G**–**I** RT-PCR analysis showed an elevated expression of NLRP3, ASC and caspase-1 mRNA in the NAc from WT mice after NAc injection of LPS, whereas these changes were attenuated in PGRNKO mice after NAc LPS treatment. *n* = 8 per group. Data were analyzed by one-way ANOVA followed by the LSD multiple comparisons test, and expressed as mean ± SEM. *p < 0.05, **p < 0.01, ***p < 0.001, ns not significant

To determine whether PGRN was a potential target for inflammatory responses and immune dysregulation in the NAc, we assessed various relevant inflammatory cytokines and molecules in the NAc from PGRNKO mice in response to NAc LPS exposure. As shown by inflammatory cytokines from ELISAs (Fig. 4A–D), NAc saline-treated PGRNKO mice exhibited slightly enhanced expression of IL-6 (*p* = 0.035) and IL-10 (*p* = 0.023) but no changes in IL-1β (*p* = 0.814) or TNF-α (*p* = 0.179) in the NAc compared to NAc saline-treated WT mice. Strikingly, PGRN deficiency reduced the increased levels of IL-1β (*p* = 0.010) and TNF-α (*p* = 0.006) in the NAc triggered by NAc injection with LPS, whereas it did not alter the elevated expression of IL-6 (*p* = 0.732) or IL-10 (*p* = 0.986) after NAc LPS injection compared to NAc LPS-treated WT mice. These results demonstrate that PGRN deficiency results in increased IL-6 and IL-10 expression in the NAc at baseline under normal conditions, while PGRN deficiency reversed the enhanced expression of IL-1β and TNF-α in the NAc under neuroinflammation conditions in the NAc, suggesting that PGRN deficiency results in different inflammatory cytokine patterns in the NAc that likely exert proinflammatory and anti-inflammatory actions under distinct scenarios.

Considering that brain inflammation is driven primarily by inflammatory mediators released by astrocytes and microglia [46], we determined the levels of GFAP-labeled astrocytes and Iba1-labeled microglial mRNA in the NAc from WT and PGRNKO mice 24 h after NAc LPS stimulation. We observed that GFAP-labeled astrocyte mRNA in the NAc from saline-treated PGRNKO mice was elevated when compared to that in the saline-WT mice (*p* = 0.036, Fig. 4F). Next, the results showed that astrocyte (*p* = 0.0001) and microglia (*p* = 0.001) mRNA expression in the NAc was highly elevated in NAc LPS-treated WT mice, but these alterations were abolished in NAc LPS-treated PGRNKO mice (GFAP, *p* = 0003; Iba1, *p* = 0.004; Fig. 4E, F). Furthermore, we assessed NLRP3 inflammasome mRNA expression in the NAc in NAc LPS-treated PGRNKO mice, a key component of the innate immune system that promotes proinflammatory cytokine IL-1β release. The results showed elevated expression of NLRP3 (*p* = 0.0001), ASC (*p* = 0.0004) and caspase-1 (*p* = 0.0001) mRNA in the NAc from WT mice after NAc injection with LPS, whereas these changes were attenuated in PGRNKO mice after NAc LPS treatment (NLRP3, *p* = 0.001; ASC, *p* = 0.002; caspase-1, *p* = 0.006; Fig. 4G–I).

Together, our results imply that PGRN deficiency seems to increase astrocyte activity and expression in the NAc, as well as dysfunction in inflammatory cytokine production, under normal circumstances, thus being involved in FTD-like behaviors; however, PGRN deficiency attenuates NAc neuroinflammatory reactions under acute immune challenge, thus resulting in the antidepressant effects in the NAc neuroinflammation-related depressive-like phenotype.

### PGRN deficiency influences NAc neuroplasticity under the conditions of NAc neuroinflammation

**Fig. 5:**
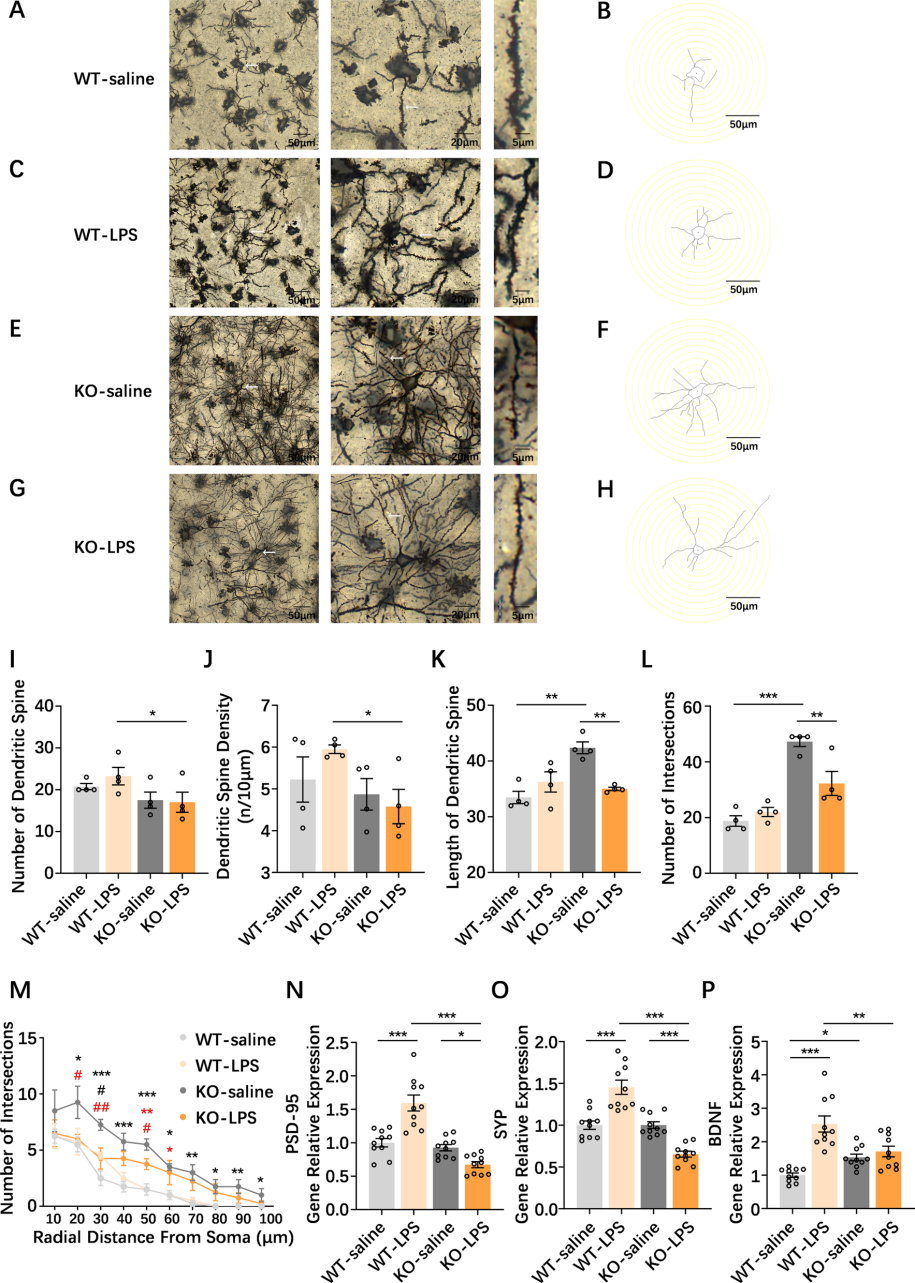
PGRN deficiency influences NAc neuroplasticity in the conditions of normal baseline and NAc neuroinflammation. **A**, **B** Representative image of a Golgi-stained dendrite in the NAc in NAc saline-treated WT mice. **C**, **D** Representative image of a Golgi-stained dendrite in WT mice NAc after NAc LPS injection. **E**, **F** Representative image of a Golgi-stained dendrite in the NAc in NAc saline-treated PGRNKO mice. **G**, **H** Representative image of a Golgi-stained dendrite in PGRNKO mice NAc during LPS exposure. Scale bar 50 µm, 20 µm, and 5 µm in sequence (**A**–**H**). **I** Sholl analysis showed that PGRN deficiency in mice decreased the number of dendritic spines in the NAc after NAc LPS challenge, compared to NAc LPS-treated WT mice. **J** Sholl analysis showed that PGRN deficiency in mice significantly decreased the dendritic spine density in the NAc following LPS injection relative to NAc LPS-treated WT mice. **K** Sholl analysis showed that the length of dendritic spine presented in the NAc in PGRNKO saline-treated mice was obviously longer than that in WT saline-treated mice. After NAc LPS challenge, the alteration in the length of dendritic spine induced by PGRN deficiency was abrogated in the NAc. **L**, **M** Sholl analysis showed that the dendritic arborization (number of intersections) in the PGRNKO saline-treated mice was significantly increased, compared to that in WT saline-treated mice. After NAc LPS challenge, the enhancement in dendritic arborization induced by PGRN deficiency was reversed in the NAc. *n* = one neuron per mouse, 3–4 mice per group (**I**–**M**). **N**–**P** RT-PCR analysis showed that synaptic marker proteins PSD-95 (**N**), SYP (**O**) and BDNF (**P**) mRNA levels induced by NAc LPS treatment were increased in the WT mice as compared to WT control mice, which were abrogated in the NAc LPS-treated PGRNKO mice. *n* = 10 per group. Data were analyzed by one-way ANOVA or two-way ANOVA followed by the LSD multiple comparisons test, and expressed as mean ± SEM. In **M**, black * means WT-saline versus KO-saline; red * means WT-LPS versus KO-LPS; black # means WT-LPS versus KO-LPS; red # means KO-saline versus KO-LPS. *p < 0.05, **p < 0.01, ***p < 0.001, ^#^*p* < 0.05, ^##^*p* < 0.01

Increasing evidence demonstrates that neuroplasticity, a fundamental mechanism of neuronal adaptation, is disrupted in patients with mood disorders and in animal models of stress [47]. Here, we tested NAc neuroplasticity, including structural plasticity (such as plasticity changes in spine and dendrite morphology) and functional synaptic plasticity (such as PSD-95, SYP and BDNF), in WT and PGRNKO mice in the context of NAc LPS stimulation-induced NAc inflammatory responses. Regarding dendrite complexity, we discovered a trend toward an increase in dendritic arborization and the number, length and spine density of dendrites occurring in the NAc in NAc LPS-treated mice compared with NAc saline-treated mice, although such changes were not significantly different after NAc inflammation exposure (Fig. 5A–D). However, PGRN deficiency attenuated the increase in the number of dendrites (*p* = 0.039; Fig. 5I–L) and dendritic spine density (*p* = 0.017; Fig. 5J) in the NAc induced by NAc LPS challenge but did not alter the differences in the NAc neuroinflammation-induced length of dendrites (Fig. 5K) or the arborization (Fig. 5L, M) of dendrites in the NAc compared to WT mice after NAc LPS injection (Fig. 5G, H). Interestingly, dendritic arborization (*p* = 0.0001; Fig. 5L, M) and length (*p* = 0.001; Fig. 5K) in the NAc were substantially enhanced in saline-treated PGRNKO mice relative to saline-treated WT mice under normal conditions (Fig. 5E, F), but in response to NAc neuroinflammation after LPS exposure, PGRN deficiency-induced dendritic alterations in the NAc, such as dendritic arborization (*p* = 0.002) and length (*p* = 0.005), were alleviated. Collectively, these data demonstrate that PGRN deficiency results in a significant enhancement in the length and connectivity of dendrites in the NAc at baseline; however, under conditions of NAc neuroinflammation, PGRN deficiency not only remarkably reduces a slightly increased tendency in NAc dendritic complexity after neuroinflammation stimulation in the NAc, but also attenuates the changes in NAc dendritic complexity induced by PGRN deficiency alone.

In addition, the results observed from functional synaptic marker proteins revealed that NAc LPS treatment-induced PSD-95 (*p* = 0.0001), SYP (*p* = 0.0001) and BDNF (*p* = 0.0001) mRNA increased in the WT mice compared to WT control mice (Fig. 5N–P), which was abrogated in NAc LPS-treated PGRNKO mice (PSD-95, *p* = 0.0001; SYP, *p* = 0.000; BDNF, *p* = 0.001; Fig. 5N–P). Strikingly, we found that BDNF mRNA expression was slightly higher in NAc saline-treated PGRNKO mice than in saline-treated WT mice. These results indicate that PGRN deficiency is sufficient to increase synaptic protein mRNA levels in the NAc at baseline in normal conditions, but PGRN deficiency alleviates the increase in NAc LPS stimulation-evoked functional synaptic protein expression in the NAc.

Taken together, our results show that PGRN deficiency increased NAc dendritic complexity and synaptic plasticity under normal conditions, but in the NAc inflammatory response, PGRN deficiency alleviated NAc LPS injection and its own mutation-induced enhancement of NAc dendritic complexity and synaptic plasticity.

### PGRN deficiency alters the p38 and NF-κB signaling pathways triggered by NAc neuroinflammation

**Fig. 6:**
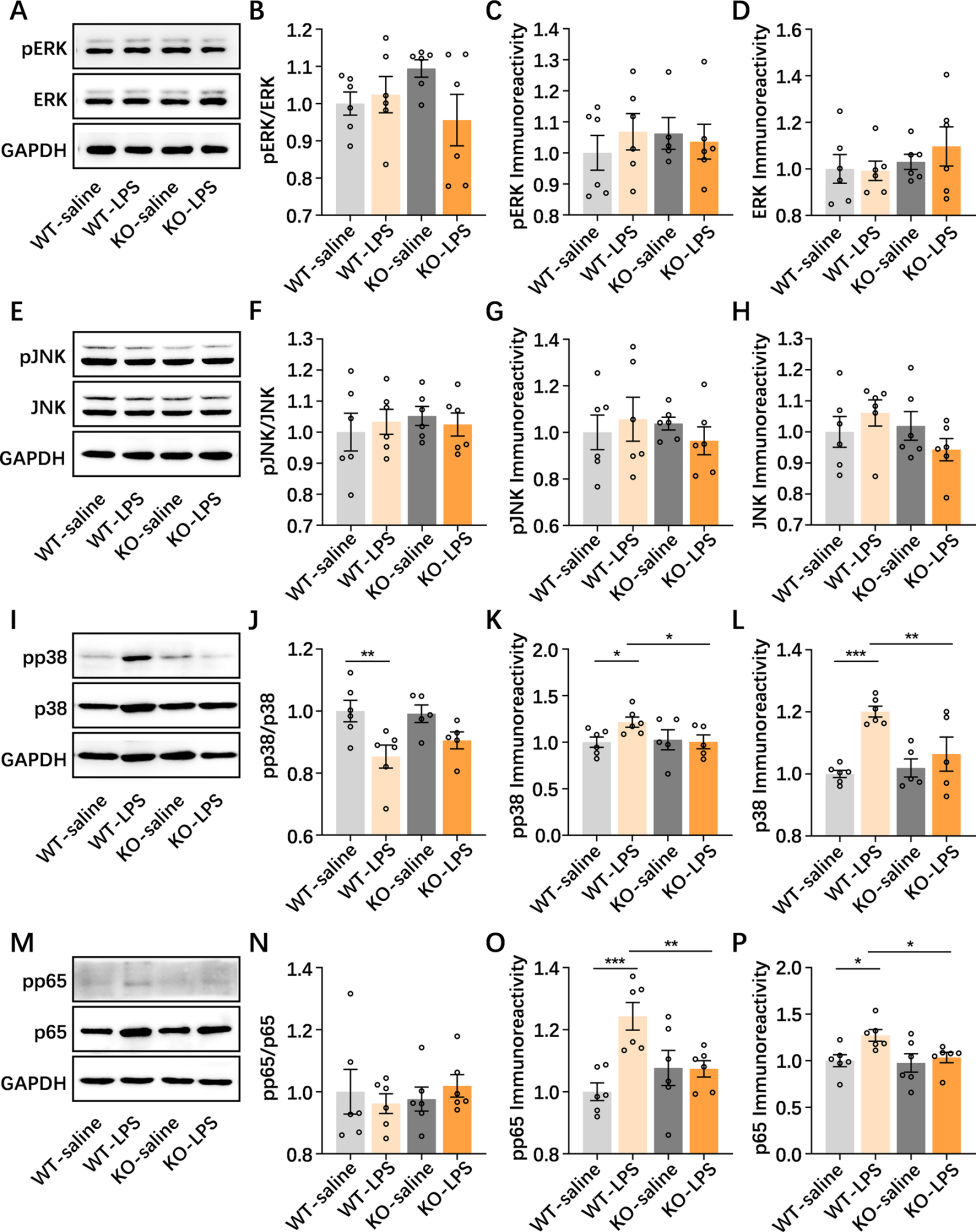
Effects of p38 and NF-κB signaling pathways on PGRN deficiency during NAc neuroinflammation. **A**–**D** Western blot analysis showed that ERK MAPK signaling pathway had no effects in the WT and PGRNKO mice after NAc LPS challenge. **E–H** Western blot analysis showed that JNK MAPK signaling pathway did not alter between WT and PGRNKO mice after NAc LPS injection. **I**–**L** The expression of p38 and p-p38 were increased and activation of p38 (p-p38/p38) was decreased in the NAc after NAc LPS injection as compared to that in NAc saline-treated mice, but these changes in p38 and p-p38 expression without p38 activation in the NAc were abolished in NAc LPS-treated PGRNKO mice. **M**–**P** The expression in p-p65 and p65 but no p65 activation (p-p65/p65) in the NAc was obviously elevated in the NAc LPS-treated WT mice relative to control mice, which was reversed in the NAc LPS-treated PGRNKO mice. *n* = 6 per group. Data were analyzed by one-way ANOVA followed by the LSD multiple comparisons test, and expressed as mean ± SEM. *p < 0.05, **p < 0.01

Neuroinflammatory reactions and synaptic plasticity mechanisms are intimately associated with the activation of the mitogen-activated protein kinase (MAPK) and nuclear factor-κB (NF-κB) signaling pathways [47, 48]. Thus, we attempted to explore whether MAPK signaling, including ERK, p38 and JNK signaling, as well as NF-κB signaling were involved in PGRN deficiency-linked NAc neuroinflammation (Additional files 1, 2, 3, 4). In the activation and expression of the ERK (Fig. 6A–D) and JNK (Fig. 6E–H) signaling pathways expressed in the NAc, no abnormalities were observed in WT or PGRNKO mice after NAc saline or LPS treatment. However, the expression of p38 (*p* = 0.0001) and p-p38 (*p* = 0.033) was increased and the activation of p38 (p-p38/p38) (*p* = 0.004) was decreased in the NAc after NAc LPS injection compared to NAc saline treatment, but these changes in p38 (*p* = 0.005) and p-p38 (*p* = 0.046) expression, but not p38 activation (*p* = 0.282), in the NAc were abolished in NAc LPS-treated PGRNKO mice (Fig. 6I–L). The results of NF-κB signaling showed that the expression of p-p65 (*p* = 0.0004) and p65 (*p* = 0.028) without the activation of p65 (p-p65/p65) (*p* = 0.574) occurring in the NAc was obviously elevated in NAc LPS-treated WT mice relative to control mice, which was reversed in LPS-treated PGRNKO mice (p-p65 expression, *p* = 0.008; p65 expression, *p* = 0.049; Fig. 6M–P). Taken together, our results demonstrated that the p38 and NF-κB signaling pathways are involved in the NAc neuroinflammation induced by LPS injection and the depressive-like phenotype; more importantly, the p38/NF-κB signaling cascade is a potential mechanism whereby PGRN deficiency exerts anti-inflammatory and antidepressant actions and alters neuroplasticity in the NAc in a mouse model of NAc LPS injection-triggered depressive-like behaviors.

## Discussion

It was recently well identified that loss-of-function mutations in the progranulin (PGRN) gene are the crucial etiology of frontotemporal dementia (FTD), which is the second most common dementia in people under the age of 65 after Alzheimer’s disease [49, 50], and subsequently induce FTD-like behavioral and neuropsychiatric disturbances in memory and learning cognition, social interaction, depressive-like and anxiety-like symptoms both in patients with genetic FTD [51] and in a mouse model of PGRN deficiency [38]. Consistent with previous findings, at 2 months, the PGRNKO mice used in our current research displayed short-term, long-term and spatial learning and memory deficits in the novel objective recognition and Morris water maze tests, impaired social interaction both in same- and different-sex social interaction tests, and an increase in disinhibition-like symptoms in the elevated plus maze test. In contrast, we did not observe any alterations in depressive-like behaviors in 2-month-old PGRNKO mice, which is different from previous reports in more than 4-month-old PGRNKO mice [38]. Notably, a recent longitudinal cohort study revealed that the depression-like symptoms observed in FTD patients associated with *GRN* gene variation significantly increased in the early stages of disease, gradually decreased in the intermediate stage and subsequently increased again in the late stage, suggesting that the progression and severity of depressive-like symptoms may change over the course of genetic FTD characterized by *GRN* variants [51]. In this context, testing ages may be a reason for these observed inconsistent behavioral performances in depressive-like behaviors in PGRNKO mice, as it has been found that PGRNKO mice at different developmental periods present diverse behaviors, with older mice with PGRN deficiency expressing more changes in depressive disorders than young mice.

In the brain, PGRN is preferentially expressed by microglia and neurons [52]. Further studies showed that PGRN was intensely immunoreactive in neurons of the cingulate and piriform cortex, hippocampus, amygdala, hypothalamus and cerebellar Purkinje cells [53]. By comparison, PGRN in microglia was not prominent under normal circumstances, while upregulated microglial PGRN expression has been discovered after various injuries, such as traumatic brain injury, toxin-induced brain injuries, spinal cord injury and nerve axotomy [32, 54–57]. Here, we found that PGRN protein was abundantly expressed in neurons of the NAc under normal conditions; the injection of LPS into the NAc resulted in the elevation of PGRN expression in the NAc, and this NAc neuroinflammation resulted in a large increase in the expression of PGRN in microglia but obviously less neuronal PGRN expression. Strikingly, astrocytes have also been implicated in the PGRN-mediated neuropathological phenotype, although PGRN is primarily expressed in microglia and neurons. PGRN has been found in lysosome-associated membrane glycoprotein (LAMP-1)-positive astrocyte vesicles [58]. Furthermore, aged PGRNKO mice and symptomatic carriers of *GRN* mutations causing FTD showed prominent astrogliosis [59, 60]. Consistently, we observed elevated mRNA expression of GFAP, a marker of astrocytes, in the NAc in PGRNKO mice; LPS-induced NAc neuroinflammation resulted in significantly increased PGRN expression in astrocytes in the NAc relative to saline controls in WT mice. Collectively, these data demonstrated that PGRN is primarily expressed in NAc neurons under normal conditions; elevated expression of PGRN was distributed in astrocytes and microglia from the NAc region after the injection of LPS into the NAc, suggesting that PGRN expressed in astrocytes and microglia in the NAc may cooperate to be involved in NAc neuroinflammation development.

PGRN has been considered a unique risk protein that sits atop the regulating cascade controlling brain health, and PGRN dysfunction is an important cause of several neurodegenerative diseases through the resulting dysregulation of neuronal survival and function and the autophagy–lysosomal pathway as well as neuroinflammation [34, 61]; however, the precise mechanisms underlying the role of PGRN in neurodegenerative diseases are still not fully known. In the current study, 2-month-old PGRNKO mice developed an FTD-like pattern of behavioral disturbances. Importantly, we found that mice with PGRN deficiency displayed mild NAc neuroinflammatory responses, showing the elevated IL-6 and IL-10 levels and GFAP mRNA expression in the NAc. In the same direction, a previous study has shown that circulating IL-6 was increased in PGRN-mutated FTD patients as compared to both PGRN non-mutated FTD patients and controls; also, in vitro study verified by IL-10 neutralizing antibody indicated that macrophages from PGRNKO mice contribute to hyperinflammatory phenotype through affecting 1L-10 production [62, 63]. These results support the potential specific role of IL-6 and IL-10 in influencing symptom occurrence in PGRN mutation-associated FTD. In addition, PGRNKO mice showed an apparent neuroplasticity increase in the NAc, namely, a significant enhancement in the length and connectivity of dendrites and BDNF expression from the NAc. Compared to the atrophy and loss of hippocampal and cortical neurons in neurodegenerative diseases [33, 64, 65], dendritic and synaptic plasticity of NAc neurons was found to be increased in mice with PGRN deficiency-associated FTD-like behaviors. In this regard, an important discovery that may contribute to explaining this phenomenon is that PGRN deficiency resulted in excessive NF-κB activation in microglia and elevated TNF-α signaling, subsequently leading to the hyperexcitability of medium spiny neurons in the NAc [66]. Furthermore, the action of BDNF is based on its involvement in synaptic plasticity via the enhancement of dendritic arborization, axonal growth, and neurotransmitter release [67]. Thus, the increased dendritic and synaptic plasticity in the NAc seems to be attributed to increased expression of BDNF occurring in PGRNKO mice. In addition to the BDNF expression and neuroplasticity changes in the NAc observed in PGRNKO mice, we found that the levels of the astrocyte and inflammatory cytokines IL-6 and IL-10 were enhanced in the NAc these mice, suggesting that PGRN deficiency results in astrocyte activation and inflammatory responses in the NAc, which in turn results in NAc neuroplasticity alterations and FTD-like behaviors. These preliminary results of our study are hypothesis generating and of interest for future studies, which would be desirable for further confirmation of our findings.

The NAc and its roles in depressive disorders have been the focus of significant research. Importantly, advanced studies have proposed that neuroinflammation occurring in the NAc is implicated in the progression of depression [14, 68], and our published studies further indicated that NAc neuroinflammation after immune challenge was sufficient to induce depressive-like behaviors in mice [16, 46]. Here, we attempted to identify the roles and functions of PGRN in the NAc neuroinflammation-linked depressive-like phenotype, mainly because some in vitro and in vivo studies showed that PGRN plays protective and anti-inflammatory roles in response to brain injury [56, 69, 70], and the gene expression of PGRN in the NAc observed in our present study was upregulated in LPS-induced NAc neuroinflammation. Unexpectedly, the neuroinflammation-induced depressive-like phenotype in the NAc was attenuated in PGRNKO mice, whereas selectively restoring recombinant PGRN in the NAc of PGRNKO mice predisposed PGRNKO mice to produce depressive-like behaviors under conditions of neuroinflammation in the NAc, indicating that PGRN present in the NAc contributes to depressive-like behaviors under conditions of NAc neuroinflammatory stimulation. In addition, GFAP-marked astrocytes and Iba1-marked microglia were increased, with corresponding elevations in the NLRP3 inflammasome and inflammatory cytokines TNF-α and IL-1β, and these changes were abolished in PGRNKO mice under conditions of NAc neuroinflammation elicited by LPS challenge. Furthermore, the neuroinflammatory-induced increase in dendritic complexity and synaptic protein expression in NAc neurons was reversed in PGRNKO mice; more importantly, PGRN deficiency triggered a significant increase in the length and connectivity of dendrites, and elevated BDNF expression in the NAc was attenuated in PGRNKO mice under conditions of NAc neuroinflammation. Based on this evidence, our present results showed that PGRN deficiency exerts antidepressant effects at least partially by attenuating astrocyte- and microglial activation-associated inflammatory responses and neuroplasticity alterations occurring in the NAc in the NAc neuroinflammation-induced depressive-like phenotype, which is opposite of the findings of other studies on brain injuries. There are some reasons to explain this divergence. First, it should be noted that chronic unpredictable stress or repeated restraint reduced dendritic complexity and spine density in the rodent prefrontal cortex and hippocampus; in contrast, the dendritic complexity of medium spiny neurons of the NAc was increased by stress exposure in rodent models [71–73]. In this light, the brain region of the NAc, with a complex neuroanatomical connectivity pattern resulting from integrating different excitatory and inhibitory inputs [74], produces totally distinct functions in the context of brain immune activation when compared with other brain nuclei, such as the hippocampus, which is not unexpected. Furthermore, an important in vitro study of PGRN expression in human brain-derived cells showed different mechanisms of regulation of PGRN between microglia and astrocytes, showing that the TLR ligands (LPS) and the proinflammatory/Th1 cytokines (IL-1β/IFN-γ) suppressed microglial PGRN, whereas they increased PGRN production from astrocytes [75]. Therefore, one complex biological activity involving PGRN in astrocytes and microglia is likely to occur in the condition of the NAc neuroinflammatory response and possibly result in surprising outcomes, but further investigation is needed. Finally, PGRN is a complex protein that may have pro- or anti-inflammatory properties depending upon the extent of PGRN-regulated proteolysis and the generation of proinflammatory GRN peptides [76]. In cultures of primary human microglia, LPS-increased PGRN cleavage occurs intracellularly, resulting in PGRN production being suppressed while GRN production is increased and switching the role of PGRN in microglia from neurotrophic to inflammatory under inflammatory conditions [75], which is consistent with our evidence of the actions of PGRN in a mouse model of NAc neuroinflammation. In summary, our findings highlight the peculiarity of the function of PGRN in response to NAc immune activation-induced depressive-like behaviors, as observed in PGRNKO mice at the cellular, molecular and behavioral levels.

Given that the MAPK and NF-κB pathways have widely been considered classic signaling pathways in inflammation, resulting in the upregulated expression of proinflammatory genes [77], but PGRN has also been reported to play a significant role in modulating NF-κB activation and MAPK signaling [78, 79]. Our results identified that the neuroinflammation-induced p38 MAPK and NF-κB signaling pathway levels were increased in the NAc, but such alterations were abolished in PGRNKO mice under neuroinflammatory conditions. These results suggest that the p38 and NF-κB pathways are involved in the antidepressant effects of PGRN deficiency on NAc neuroinflammation-promoted depressive-like behaviors. Our present study has some limitations. Considering that short- and long-term neuroinflammation-induced depression have distinct regulatory mechanisms, it is still unclear whether the anti-inflammatory roles occurring in the NAc and the antidepressant effects after PGRN deficiency could be induced in other animal models of depression, such as models of chronic stress exposure. In addition, astrocyte and microglial PGRN present in the NAc seem to play different roles in the pattern of NAc immune activation according to our observations, and it will be of great interest to investigate the potential regulatory mechanisms by which PGRN occurs in distinct cell-type patterns in the NAc in response to NAc neuroinflammation and subsequently modulates neural plasticity and depressive disorders. 

## Conclusion

**Fig. 7:**
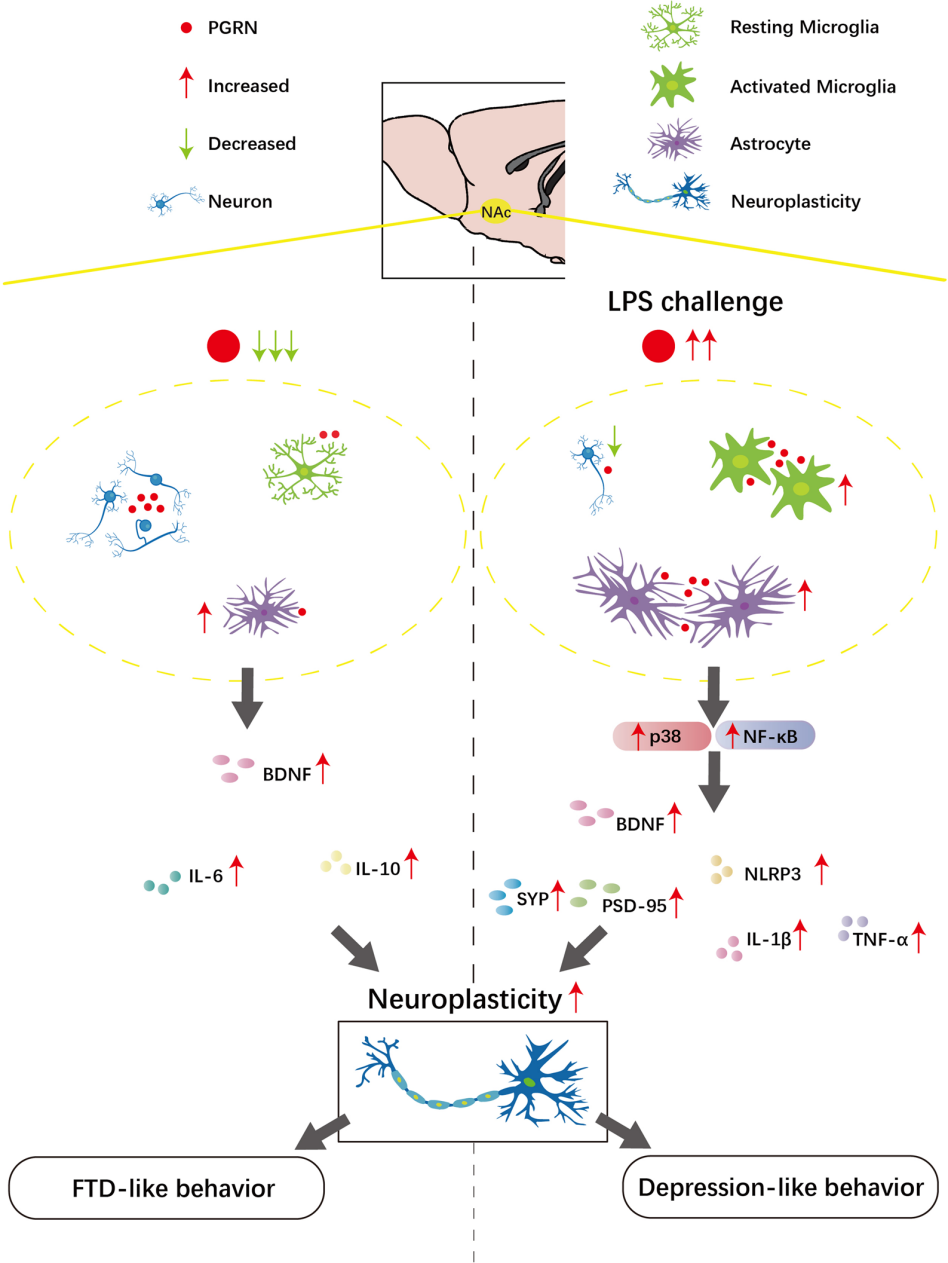
Working model of PGRN functions presented in the NAc in FTD-like behavior and depression-like behavior. We show that PGRN deficiency-induced FTD-like behavioral deficits correlates with astrocyte activation, neuroinflammatory cytokines disturbances and neuroplasticity increase observing in the NAc. In the NAc neuroinflammation-triggered depressive-like mouse model, PGRN stimulates astrocytes and microglial activation in the NAc, thus promoting neuroinflammatory reaction and neuroplasticity increase presented in the NAc and subsequent depressive-like behaviors via p38 and NF-κB pathways

Our results reveal that PGRN deficiency-induced FTD-like behavioral and neuropsychiatric deficits correlate with the astrocyte activation, neuroinflammatory cytokine disturbances and neuroplasticity increases observed in the NAc. Notably, PGRN stimulates astrocyte and microglial activation in the NAc under acute immune challenge, thus promoting neuroinflammatory reactions and neuroplasticity increases in the NAc and subsequent depressive-like behaviors via the p38 and NF-κB pathways (Fig. 7). Thus, a better understanding of the roles of PGRN present in the NAc at distinct states may open novel therapeutic avenues for treating neuropsychiatric disorders.

## Supplementary Information

**Additional file 1: Figure S1.** The original western blot image of ERK MAPK signaling pathway.

**Additional file 2: Figure S2.** The original western blot image of JNK MAPK signaling pathway.

**Additional file 3: Figure S3.** The original western blot image of p38 MAPK signaling pathway.

**Additional file 4: Figure S4.** The original western blot image of NF-κB signaling pathway.

## Data Availability

All data are available from the corresponding author upon reasonable request.

## Abbreviations

NAc: Nucleus accumbens
PGRN: Progranulin
WT: Wild type
KO: Knockout
LPS: Lipopolysaccharide
FTD: Frontotemporal dementia
MSNs: Medium spiny neurons
mEPSC: Miniature excitatory post-synaptic currents
D1: Dopamine D1 receptor
D2: Dopamine D2 receptor
GRN: Granulins
MAPK: Mitogen-activated protein kinase
ERK1/2: Extracellular regulated protein kinase 1 and 2
NF-κB: Nuclear factor of kappa
JNK: C-Jun N-terminal kinase
JAK/STAT: Janus kinase/signal transducer and activator of transcription
Th1: T-helper 1
Th17: T-helper 17
Foxo4/STAT3: Forkhead box protein O4/signal transducer and activator of transcription 3
IL: Interleukin
TNF-α: Tumor necrosis factor-alpha
FST: Forced swim test
TST: Tail suspension test
SPT: Sucrose preference test
AP: Anterior posterior
ML: Medial lateral
DV: Dorsal ventral
LAMP-1: Lysosome-associated membrane glycoprotein

## References

[1] Malhi GS, Mann JJ (2018). Depression. Lancet.

[2] Tang F, Liang J, Zhang H, Kelifa MM, He Q, Wang P (2021). COVID-19 related depression and anxiety among quarantined respondents. Psychol Health.

[3] Russo SJ, Nestler EJ (2013). The brain reward circuitry in mood disorders. Nat Rev Neurosci.

[4] Heshmati M, Russo SJ (2015). Anhedonia and the brain reward circuitry in depression. Curr Behav Neurosci Rep.

[5] Satterthwaite TD, Kable JW, Vandekar L, Katchmar N, Bassett DS, Baldassano CF (2015). Common and dissociable dysfunction of the reward system in bipolar and unipolar depression. Neuropsychopharmacology.

[6] Pizzagalli DA, Holmes AJ, Dillon DG, Goetz EL, Birk JL, Bogdan R (2009). Reduced caudate and nucleus accumbens response to rewards in unmedicated individuals with major depressive disorder. Am J Psychiatry.

[7] Anacker C, Scholz J, O'Donnell KJ, Allemang-Grand R, Diorio J, Bagot RC (2016). Neuroanatomic differences associated with stress susceptibility and resilience. Biol Psychiatry.

[8] Schlaepfer TE, Cohen MX, Frick C, Kosel M, Brodesser D, Axmacher N (2008). Deep brain stimulation to reward circuitry alleviates anhedonia in refractory major depression. Neuropsychopharmacology.

[9] Bewernick BH, Hurlemann R, Matusch A, Kayser S, Grubert C, Hadrysiewicz B (2010). Nucleus accumbens deep brain stimulation decreases ratings of depression and anxiety in treatment-resistant depression. Biol Psychiatry.

[10] Francis TC, Lobo MK (2017). Emerging role for nucleus accumbens medium spiny neuron subtypes in depression. Biol Psychiatry.

[11] Francis TC, Chandra R, Friend DM, Finkel E, Dayrit G, Miranda J (2015). Nucleus accumbens medium spiny neuron subtypes mediate depression-related outcomes to social defeat stress. Biol Psychiatry.

[12] Harrison NA, Brydon L, Walker C, Gray MA, Steptoe A, Critchley HD (2009). Inflammation causes mood changes through alterations in subgenual cingulate activity and mesolimbic connectivity. Biol Psychiatry.

[13] Menard C, Pfau ML, Hodes GE, Kana V, Wang VX, Bouchard S (2017). Social stress induces neurovascular pathology promoting depression. Nat Neurosci.

[14] Décarie-Spain L, Sharma S, Hryhorczuk C, Issa-Garcia V, Barker PA, Arbour N (2018). Nucleus accumbens inflammation mediates anxiodepressive behavior and compulsive sucrose seeking elicited by saturated dietary fat. Mol Metab.

[15] Wang J, Jia Y, Li G, Wang B, Zhou T, Zhu L (2018). The Dopamine receptor D3 regulates lipopolysaccharide-induced depressive-like behavior in mice. Int J Neuropsychopharmacol.

[16] Wang J, Lai S, Wang R, Zhou T, Dong N, Zhu L (2022). Dopamine D3 receptor in the nucleus accumbens alleviates neuroinflammation in a mouse model of depressive-like behavior. Brain Behav Immun.

[17] He Z, Ismail A, Kriazhev L, Sadvakassova G, Bateman A (2002). Progranulin (PC-cell-derived growth factor/acrogranin) regulates invasion and cell survival. Cancer Res.

[18] He Z, Ong CH, Halper J, Bateman A (2003). Progranulin is a mediator of the wound response. Nat Med.

[19] Tang W, Lu Y, Tian QY, Zhang Y, Guo FJ, Liu GY (2011). The growth factor progranulin binds to TNF receptors and is therapeutic against inflammatory arthritis in mice. Science.

[20] Chen J, Li S, Shi J, Zhang L, Li J, Chen S (2016). Serum progranulin irrelated with Breg cell levels, but elevated in RA patients, reflecting high disease activity. Rheumatol Int.

[21] Thurner L, Stöger E, Fadle N, Klemm P, Regitz E, Kemele M (2014). Proinflammatory progranulin antibodies in inflammatory bowel diseases. Dig Dis Sci.

[22] Huang K, Chen A, Zhang X, Song Z, Xu H, Cao J (2015). Progranulin is preferentially expressed in patients with psoriasis vulgaris and protects mice from psoriasis-like skin inflammation. Immunology.

[23] Pogonowska M, Poniatowski ŁA, Wawrzyniak A, Królikowska K, Kalicki B (2019). The role of progranulin (PGRN) in the modulation of anti-inflammatory response in asthma. Cent Eur J Immunol.

[24] Tian Q, Zhao Y, Mundra JJ, Gonzalez-Gugel E, Jian J, Uddin SM (2014). Three TNFR-binding domains of PGRN act independently in inhibition of TNF-alpha binding and activity. Front Biosci.

[25] Tian Q, Zhao S, Liu C (2014). A solid-phase assay for studying direct binding of progranulin to TNFR and progranulin antagonism of TNF/TNFR interactions. Methods Mol Biol.

[26] Li L, Li L, Xiao L, Shangguan J (2018). Progranulin ameliorates coxsackievirus-B3-induced viral myocarditis by downregulating Th1 and Th17 cells. Exp Cell Res.

[27] Fu W, Hu W, Shi L, Mundra JJ, Xiao G, Dustin ML (2017). Foxo4- and Stat3-dependent IL-10 production by progranulin in regulatory T cells restrains inflammatory arthritis. FASEB J.

[28] Hemmi H, Takeuchi O, Kawai T, Kaisho T, Sato S, Sanjo H (2000). A toll-like receptor recognizes bacterial DNA. Nature.

[29] Krieg AM (2002). CpG motifs in bacterial DNA and their immune effects. Annu Rev Immunol.

[30] Zhu J, Nathan C, Jin W, Sim D, Ashcroft GS, Wahl SM (2002). Conversion of proepithelin to epithelins: roles of SLPI and elastase in host defense and wound repair. Cell.

[31] Vercellino M, Grifoni S, Romagnolo A, Masera S, Mattioda A, Trebini C (2011). Progranulin expression in brain tissue and cerebrospinal fluid levels in multiple sclerosis. Mult Scler.

[32] Petkau TL, Neal SJ, Orban PC, MacDonald JL, Hill AM, Lu G (2010). Progranulin expression in the developing and adult murine brain. J Comp Neurol.

[33] Zhang T, Du H, Santos MN, Wu X, Pagan MD, Trigiani LJ (2022). Differential regulation of progranulin derived granulin peptides. Mol Neurodegener.

[34] Rhinn H, Tatton N, McCaughey S, Kurnellas M, Rosenthal A (2022). Progranulin as a therapeutic target in neurodegenerative diseases. Trends Pharmacol Sci.

[35] Olczak M, Poniatowski ŁA, Siwińska A, Kwiatkowska M, Chutorański D, Wierzba-Bobrowicz T (2021). Elevated serum and urine levels of progranulin (PGRN) as a predictor of microglia activation in the early phase of traumatic brain injury: a further link with the development of neurodegenerative diseases. Folia Neuropathol.

[36] Cruts M, Gijselinck I, van der Zee J, Engelborghs S, Wils H, Pirici D (2006). Null mutations in progranulin cause ubiquitin-positive frontotemporal dementia linked to chromosome 17q21. Nature.

[37] Baker M, Mackenzie IR, Pickering-Brown SM, Gass J, Rademakers R, Lindholm C (2006). Mutations in progranulin cause tau-negative frontotemporal dementia linked to chromosome 17. Nature.

[38] Yin F, Dumont M, Banerjee R, Ma Y, Li H, Lin MT (2010). Behavioral deficits and progressive neuropathology in progranulin-deficient mice: a mouse model of frontotemporal dementia. FASEB J.

[39] Poos JM, van den Berg E, Papma JM, van der Tholen FC, Seelaar H, Donker Kaat L (2022). Mindfulness-based stress reduction in pre-symptomatic genetic frontotemporal dementia: a pilot study. Front Psychiatry.

[40] Zhang K, Li YJ, Feng D, Zhang P, Wang YT, Li X (2017). Imbalance between TNFα and progranulin contributes to memory impairment and anxiety in sleep-deprived mice. Sci Rep.

[41] Menzel L, Kleber L, Friedrich C, Hummel R, Dangel L, Winter J (2017). Progranulin protects against exaggerated axonal injury and astrogliosis following traumatic brain injury. Glia.

[42] Xu X, Gou L, Zhou M, Yang F, Zhao Y, Feng T (2016). Progranulin protects against endotoxin-induced acute kidney injury by downregulating renal cell death and inflammatory responses in mice. Int Immunopharmacol.

[43] Ni T, Zhu L, Wang S, Zhu W, Xue Y, Zhu Y (2022). Medial prefrontal cortex Notch1 signalling mediates methamphetamine-induced psychosis via Hes1-dependent suppression of GABA(B1) receptor expression. Mol Psychiatry.

[44] Liang M, Zhu L, Wang R, Su H, Ma D, Wang H (2022). Methamphetamine exposure in adolescent impairs memory of mice in adulthood accompanied by changes in neuroplasticity in the dorsal hippocampus. Front Cell Neurosci.

[45] Livak KJ, Schmittgen TD (2001). Analysis of relative gene expression data using real-time quantitative PCR and the 2(-Delta Delta C(T)) Method. Methods.

[46] Wang J, Lai S, Li G, Zhou T, Wang B, Cao F (2020). Microglial activation contributes to depressive-like behavior in dopamine D3 receptor knockout mice. Brain Behav Immun.

[47] Pittenger C, Duman RS (2008). Stress, depression, and neuroplasticity: a convergence of mechanisms. Neuropsychopharmacology.

[48] Su Y, Liu Y, He D, Hu G, Wang H, Ye B (2022). Hordenine inhibits neuroinflammation and exerts neuroprotective effects via inhibiting NF-κB and MAPK signaling pathways in vivo and in vitro. Int Immunopharmacol.

[49] Reifschneider A, Robinson S, van Lengerich B, Gnörich J, Logan T, Heindl S (2022). Loss of TREM2 rescues hyperactivation of microglia, but not lysosomal deficits and neurotoxicity in models of progranulin deficiency. EMBO J.

[50] Amin S, Carling G, Gan L (2022). New insights and therapeutic opportunities for progranulin-deficient frontotemporal dementia. Curr Opin Neurobiol.

[51] Benussi A, Premi E, Gazzina S, Brattini C, Bonomi E, Alberici A (2021). Progression of behavioral disturbances and neuropsychiatric symptoms in patients with genetic frontotemporal dementia. JAMA Netw Open.

[52] Kleinberger G, Capell A, Haass C, Van Broeckhoven C (2013). Mechanisms of granulin deficiency: lessons from cellular and animal models. Mol Neurobiol.

[53] Daniel R, He Z, Carmichael KP, Halper J, Bateman A (2000). Cellular localization of gene expression for progranulin. J Histochem Cytochem.

[54] Tanaka Y, Matsuwaki T, Yamanouchi K, Nishihara M (2013). Increased lysosomal biogenesis in activated microglia and exacerbated neuronal damage after traumatic brain injury in progranulin-deficient mice. Neuroscience.

[55] Naphade SB, Kigerl KA, Jakeman LB, Kostyk SK, Popovich PG, Kuret J (2010). Progranulin expression is upregulated after spinal contusion in mice. Acta Neuropathol.

[56] Martens LH, Zhang J, Barmada SJ, Zhou P, Kamiya S, Sun B (2012). Progranulin deficiency promotes neuroinflammation and neuron loss following toxin-induced injury. J Clin Invest.

[57] Beel S, Herdewyn S, Fazal R, De Decker M, Moisse M, Robberecht W (2018). Progranulin reduces insoluble TDP-43 levels, slows down axonal degeneration and prolongs survival in mutant TDP-43 mice. Mol Neurodegener.

[58] Almeida S, Zhou L, Gao FB (2011). Progranulin, a glycoprotein deficient in frontotemporal dementia, is a novel substrate of several protein disulfide isomerase family proteins. PLoS ONE.

[59] Petkau TL, Hill A, Leavitt BR (2016). Core neuropathological abnormalities in progranulin-deficient mice are penetrant on multiple genetic backgrounds. Neuroscience.

[60] Heller C, Foiani MS, Moore K, Convery R, Bocchetta M, Neason M (2020). Plasma glial fibrillary acidic protein is raised in progranulin-associated frontotemporal dementia. J Neurol Neurosurg Psychiatry.

[61] Lee WC, Almeida S, Prudencio M, Caulfield TR, Zhang YJ, Tay WM (2014). Targeted manipulation of the sortilin–progranulin axis rescues progranulin haploinsufficiency. Hum Mol Genet.

[62] Bossù P, Salani F, Alberici A, Archetti S, Bellelli G, Galimberti D (2011). Loss of function mutations in the progranulin gene are related to pro-inflammatory cytokine dysregulation in frontotemporal lobar degeneration patients. J Neuroinflammation.

[63] Yin F, Banerjee R, Thomas B, Zhou P, Qian L, Jia T (2010). Exaggerated inflammation, impaired host defense, and neuropathology in progranulin-deficient mice. J Exp Med.

[64] Raitano S, Ordovàs L, De Muynck L, Guo W, Espuny-Camacho I, Geraerts M (2015). Restoration of progranulin expression rescues cortical neuron generation in an induced pluripotent stem cell model of frontotemporal dementia. Stem Cell Rep.

[65] Minami SS, Min SW, Krabbe G, Wang C, Zhou Y, Asgarov R (2014). Progranulin protects against amyloid β deposition and toxicity in Alzheimer's disease mouse models. Nat Med.

[66] Krabbe G, Minami SS, Etchegaray JI, Taneja P, Djukic B, Davalos D (2017). Microglial NFκB-TNFα hyperactivation induces obsessive-compulsive behavior in mouse models of progranulin-deficient frontotemporal dementia. Proc Natl Acad Sci USA.

[67] Ibarra IL, Ratnu VS, Gordillo L, Hwang IY, Mariani L, Weinand K (2022). Comparative chromatin accessibility upon BDNF stimulation delineates neuronal regulatory elements. Mol Syst Biol.

[68] Wu XB, Jing PB, Zhang ZJ, Cao DL, Gao MH, Jiang BC (2018). Chemokine receptor CCR2 contributes to neuropathic pain and the associated depression via increasing NR2B-mediated currents in both D1 and D2 dopamine receptor-containing medium spiny neurons in the nucleus accumbens shell. Neuropsychopharmacology.

[69] Zheng X, Mi T, Wang R, Zhang Z, Li W, Zhao J (2022). Progranulin deficiency promotes persistent neuroinflammation and causes regional pathology in the hippocampus following traumatic brain injury. Glia.

[70] Ma Y, Matsuwaki T, Yamanouchi K, Nishihara M (2017). Progranulin protects hippocampal neurogenesis via suppression of neuroinflammatory responses under acute immune stress. Mol Neurobiol.

[71] Magariños AM, McEwen BS (1995). Stress-induced atrophy of apical dendrites of hippocampal CA3c neurons: involvement of glucocorticoid secretion and excitatory amino acid receptors. Neuroscience.

[72] Wellman CL (2001). Dendritic reorganization in pyramidal neurons in medial prefrontal cortex after chronic corticosterone administration. J Neurobiol.

[73] Krishnan V, Han MH, Graham DL, Berton O, Renthal W, Russo SJ (2007). Molecular adaptations underlying susceptibility and resistance to social defeat in brain reward regions. Cell.

[74] Krishnan V, Nestler EJ (2008). The molecular neurobiology of depression. Nature.

[75] Suh HS, Choi N, Tarassishin L, Lee SC (2012). Regulation of progranulin expression in human microglia and proteolysis of progranulin by matrix metalloproteinase-12 (MMP-12). PLoS ONE.

[76] Lan YJ, Sam NB, Cheng MH, Pan HF, Gao J (2021). Progranulin as a potential therapeutic target in immune-mediated diseases. J Inflamm Res.

[77] Wang L, Yin C, Liu T, Abdul M, Zhou Y, Cao JL (2020). Pellino1 regulates neuropathic pain as well as microglial activation through the regulation of MAPK/NF-κB signaling in the spinal cord. J Neuroinflammation.

[78] Liu L, Guo H, Song A, Huang J, Zhang Y, Jin S (2020). Progranulin inhibits LPS-induced macrophage M1 polarization via NF-кB and MAPK pathways. BMC Immunol.

[79] Sun S, Zhou J, Li Z, Wu Y, Wang H, Zheng Q (2022). Progranulin promotes hippocampal neurogenesis and alleviates anxiety-like behavior and cognitive impairment in adult mice subjected to cerebral ischemia. CNS Neurosci Ther.

