# Supplementary figures and images for: Progranulin from different gliocytes in the nucleus accumbens exerts distinct roles in FTD- and neuroinflammation-induced depression-like behaviors
Source: J Neuroinflammation. 2022 Dec 29;19:318. doi: 10.1186/s12974-022-02684-8 (PMC9798954; doi:10.1186/s12974-022-02684-8)

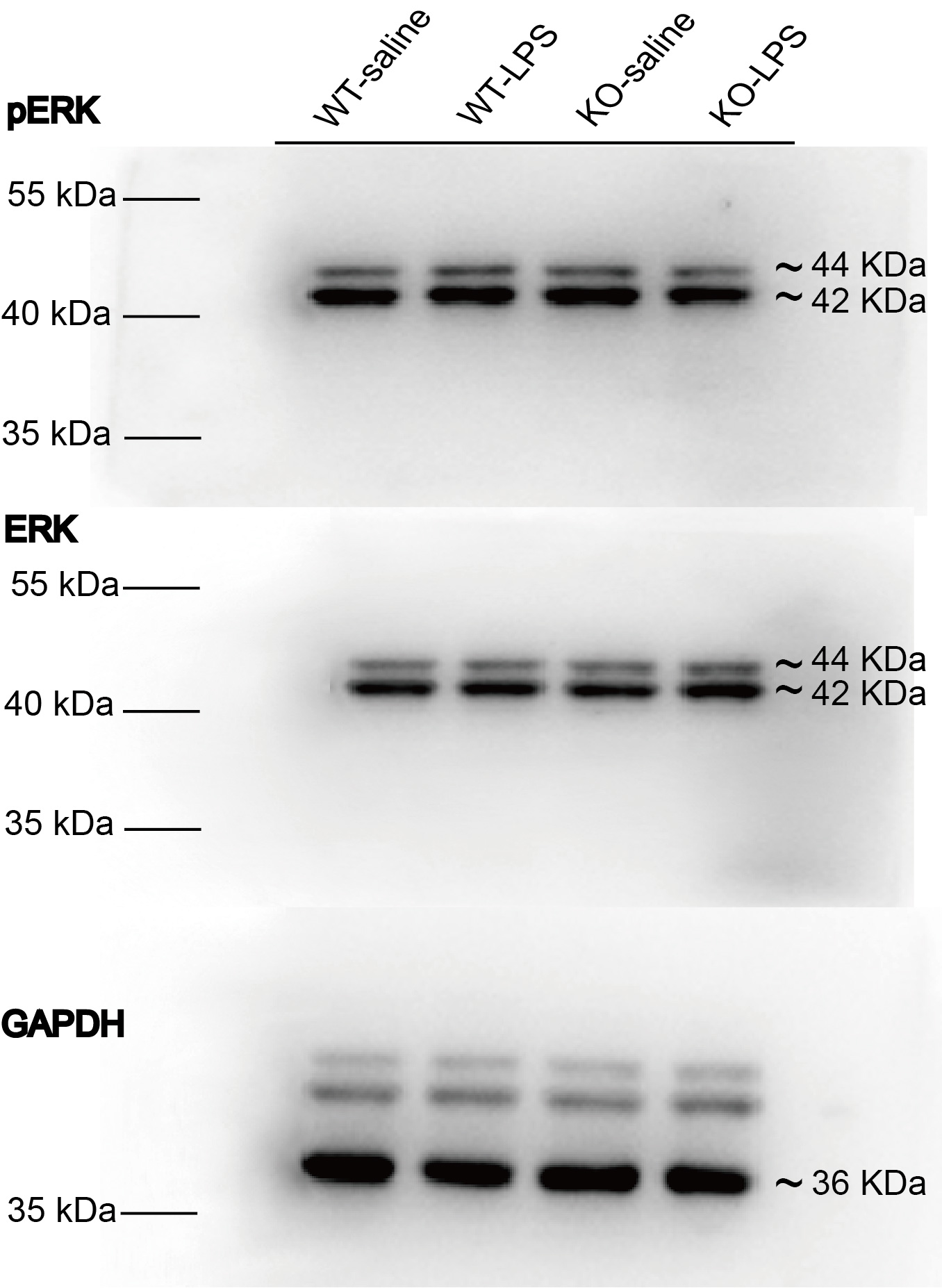

Supplement: Supplementary file 1 — Additional file 1: Figure S1. The original western blot image of ERK MAPK signaling pathway. [file 12974_2022_2684_MOESM1_ESM.jpg]

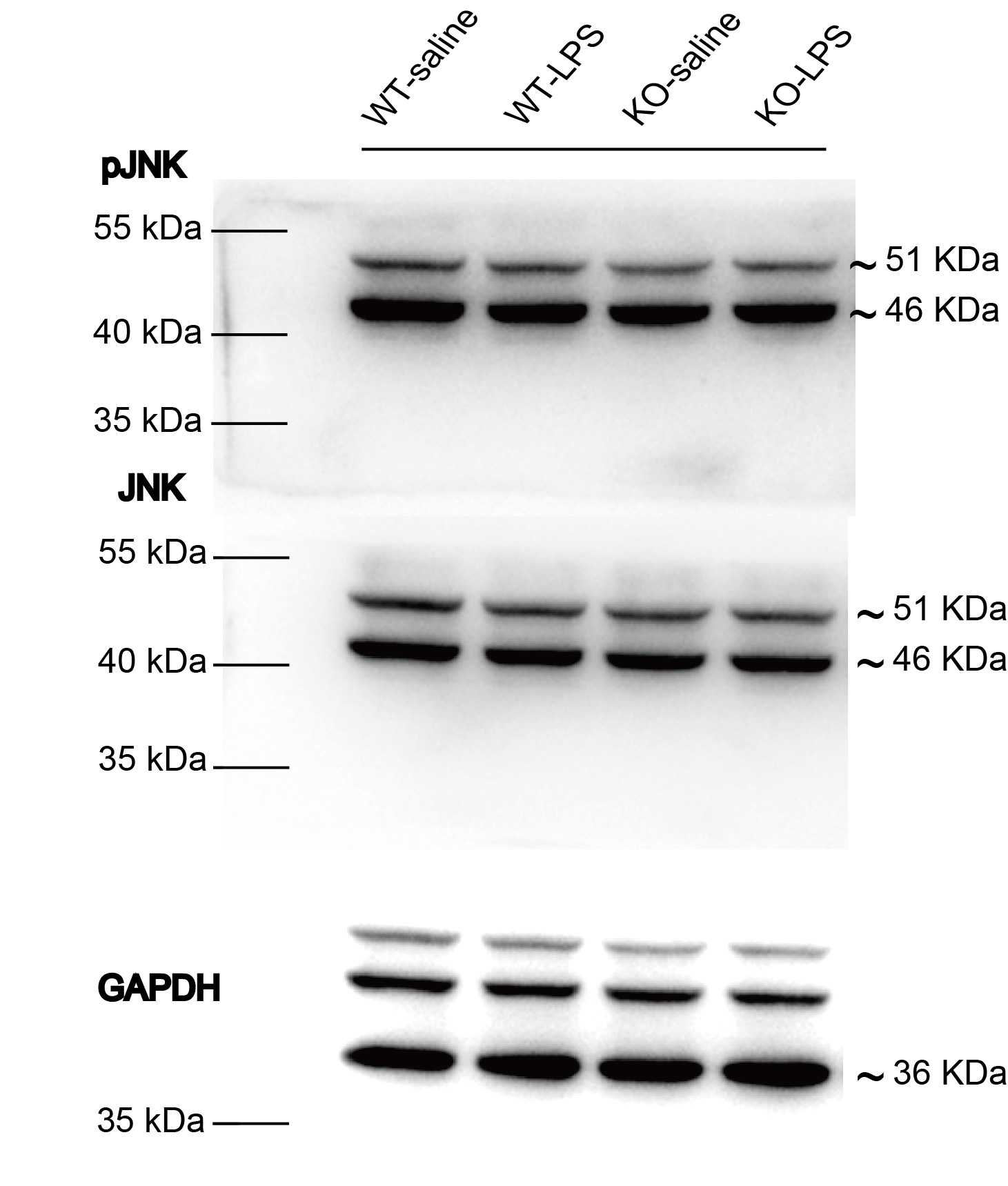

Supplement: Supplementary file 2 — Additional file 2: Figure S2. The original western blot image of JNK MAPK signaling pathway. [file 12974_2022_2684_MOESM2_ESM.jpg]

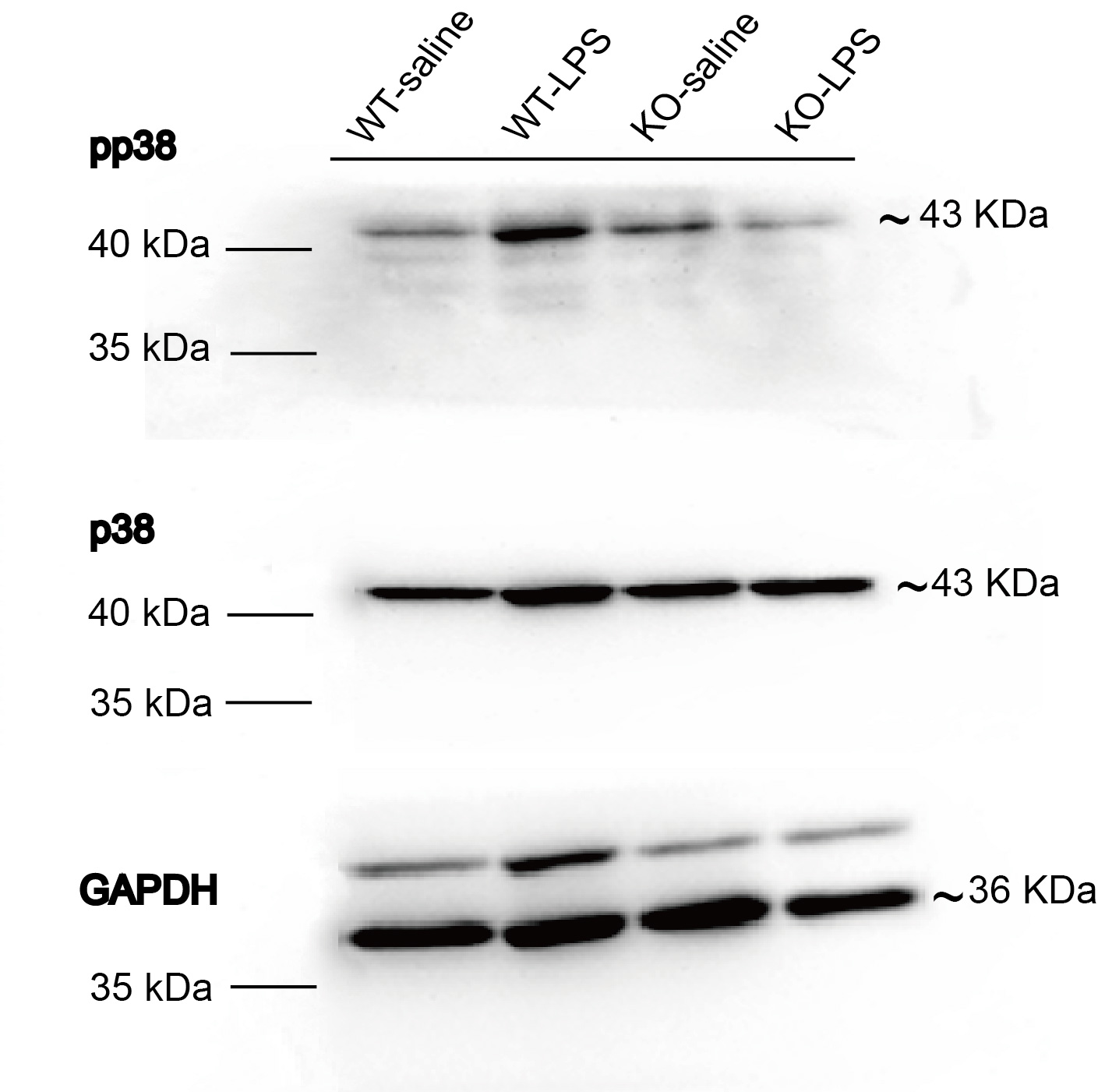

Supplement: Supplementary file 3 — Additional file 3: Figure S3. The original western blot image of p38 MAPK signaling pathway. [file 12974_2022_2684_MOESM3_ESM.jpg]

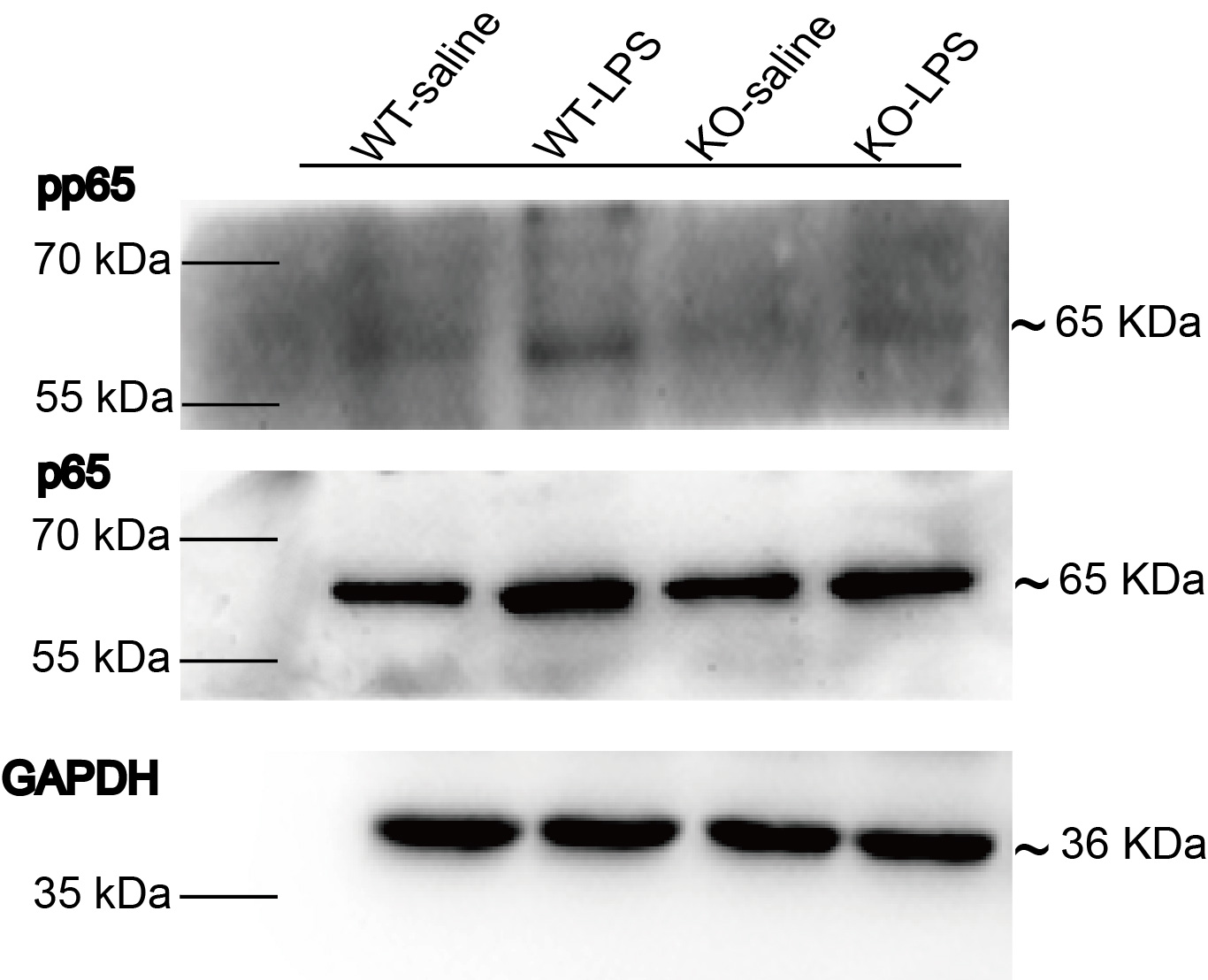

Supplement: Supplementary file 4 — Additional file 4: Figure S4. The original western blot image of NF-κB signaling pathway. [file 12974_2022_2684_MOESM4_ESM.jpg]
